# Health and economic impact of air pollution in the states of India: the Global Burden of Disease Study 2019

**DOI:** 10.1016/S2542-5196(20)30298-9

**Published:** 2020-12-27

**Authors:** India State-Level Disease Burden Initiative Air Pollution Collaborators, Anamika Pandey, Michael Brauer, Maureen L Cropper, Kalpana Balakrishnan, Prashant Mathur, Sagnik Dey, Burak Turkgulu, G Anil Kumar, Mukesh Khare, Gufran Beig, Tarun Gupta, Rinu P Krishnankutty, Kate Causey, Aaron J Cohen, Stuti Bhargava, Ashutosh N Aggarwal, Anurag Agrawal, Shally Awasthi, Fiona Bennitt, Sadhana Bhagwat, P Bhanumati, Katrin Burkart, Joy K Chakma, Thomas C Chiles, Sourangsu Chowdhury, D J Christopher, Subhojit Dey, Samantha Fisher, Barbara Fraumeni, Richard Fuller, Aloke G Ghoshal, Mahaveer J Golechha, Prakash C Gupta, Rachita Gupta, Rajeev Gupta, Shreekant Gupta, Sarath Guttikunda, David Hanrahan, Sivadasanpillai Harikrishnan, Panniyammakal Jeemon, Tushar K Joshi, Rajni Kant, Surya Kant, Tanvir Kaur, Parvaiz A Koul, Praveen Kumar, Rakesh Kumar, Samantha L Larson, Rakesh Lodha, Kishore K Madhipatla, P A Mahesh, Ridhima Malhotra, Shunsuke Managi, Keith Martin, Matthews Mathai, Joseph L Mathew, Ravi Mehrotra, B V Murali Mohan, Viswananthan Mohan, Satinath Mukhopadhyay, Parul Mutreja, Nitish Naik, Sanjeev Nair, Jeyaraj D Pandian, Pallavi Pant, Arokiasamy Perianayagam, Dorairaj Prabhakaran, Poornima Prabhakaran, Goura K Rath, Shamika Ravi, Ambuj Roy, Yogesh D Sabde, Sundeep Salvi, Sankar Sambandam, Bhavay Sharma, Meenakshi Sharma, Shweta Sharma, R S Sharma, Aakash Shrivastava, Sujeet Singh, Virendra Singh, Rodney Smith, Jeffrey D Stanaway, Gabrielle Taghian, Nikhil Tandon, J S Thakur, Nihal J Thomas, G S Toteja, Chris M Varghese, Chandra Venkataraman, Krishnan N Venugopal, Katherine D Walker, Alexandrea Y Watson, Sarah Wozniak, Denis Xavier, Gautam N Yadama, Geetika Yadav, D K Shukla, Hendrik J Bekedam, K Srinath Reddy, Randeep Guleria, Theo Vos, Stephen S Lim, Rakhi Dandona, Sunil Kumar, Pushpam Kumar, Philip J Landrigan, Lalit Dandona

## Abstract

**Background:**

The association of air pollution with multiple adverse health outcomes is becoming well established, but its negative economic impact is less well appreciated. It is important to elucidate this impact for the states of India.

**Methods:**

We estimated exposure to ambient particulate matter pollution, household air pollution, and ambient ozone pollution, and their attributable deaths and disability-adjusted life-years in every state of India as part of the Global Burden of Disease Study (GBD) 2019. We estimated the economic impact of air pollution as the cost of lost output due to premature deaths and morbidity attributable to air pollution for every state of India, using the cost-of-illness method.

**Findings:**

1·67 million (95% uncertainty interval 1·42–1·92) deaths were attributable to air pollution in India in 2019, accounting for 17·8% (15·8–19·5) of the total deaths in the country. The majority of these deaths were from ambient particulate matter pollution (0·98 million [0·77–1·19]) and household air pollution (0·61 million [0·39–0·86]). The death rate due to household air pollution decreased by 64·2% (52·2–74·2) from 1990 to 2019, while that due to ambient particulate matter pollution increased by 115·3% (28·3–344·4) and that due to ambient ozone pollution increased by 139·2% (96·5–195·8). Lost output from premature deaths and morbidity attributable to air pollution accounted for economic losses of US$28·8 billion (21·4–37·4) and $8·0 billion (5·9–10·3), respectively, in India in 2019. This total loss of $36·8 billion (27·4–47·7) was 1·36% of India's gross domestic product (GDP). The economic loss as a proportion of the state GDP varied 3·2 times between the states, ranging from 0·67% (0·47–0·91) to 2·15% (1·60–2·77), and was highest in the low per-capita GDP states of Uttar Pradesh, Bihar, Rajasthan, Madhya Pradesh, and Chhattisgarh. Delhi had the highest per-capita economic loss due to air pollution, followed by Haryana in 2019, with 5·4 times variation across all states.

**Interpretation:**

The high burden of death and disease due to air pollution and its associated substantial adverse economic impact from loss of output could impede India's aspiration to be a $5 trillion economy by 2024. Successful reduction of air pollution in India through state-specific strategies would lead to substantial benefits for both the health of the population and the economy.

**Funding:**

UN Environment Programme; Bill & Melinda Gates Foundation; and Indian Council of Medical Research, Department of Health Research, Ministry of Health and Family Welfare, Government of India.

## Introduction

Air pollution is a major cause of premature death and disease, and is the largest environmental health threat globally.^1^, ^2^, ^3^, ^4^, ^5^ Besides endangering health and shortening lifespan, air pollution adversely affects economic productivity.^6^, ^7^ The Sustainable Development Goals (SDGs) call for reduction of the burden of deaths and diseases from air pollution.^8^

Air pollution risks are typically quantified for ambient particulate matter pollution, household air pollution, and, to a smaller extent, tropospheric ozone. The main sources of ambient particulate matter pollution in India are residential and commercial biomass burning, windblown mineral dust, coal burning for energy generation, industrial emissions, agricultural stubble burning, waste burning, construction activities, brick kilns, transport vehicles, and diesel generators.^9^, ^10^, ^11^, ^12^, ^13^, ^14^, ^15^, ^16^ Household air pollution is caused mainly by the use of solid fuels for cooking, such as wood, dung, agricultural residues, coal, and charcoal.^17^, ^18^, ^19^ Ground-level ambient ozone is produced when pollutants emitted from transport vehicles, power plants, factories, and other sources react in the presence of sunlight with hydrocarbons emitted from diverse sources.^20^

Evidence of the adverse effects of air pollution on health has been growing in India.^21^ Studies from India have shown that short-term and long-term exposure are associated with disease burden and mortality.^22^, ^23^, ^24^, ^25^ The India State-Level Disease Burden Initiative has reported detailed findings on exposure to air pollution and its impacts on deaths, disease burden, and life expectancy in the states of India as part of the Global Burden of Disease Study (GBD) 2017.^21^ Improved methods and new data used in GBD 2019 have led to revised estimates of the impact of air pollution on deaths and disease burden.^26^ In this Article, we present these updated estimates for India and its states.

**Research in context**

**Evidence before the study**

Existing evidence suggests that air pollution not only affects health but also has consequences for the economy. We searched PubMed for published literature on the health and economic impacts of air pollution in India, Google for reports in the public domain, and references in these papers and reports, using the search terms “air pollutants”, “air pollution”, “ambient ozone pollution”, “ambient particulate matter pollution”, “burden”, “cost-of-illness”, “DALY”, “death”, “economic impact”, “household air pollution”, “India”, “indoor pollution”, “morbidity”, “mortality”, and “PM_2·5_ exposure”, on June 12, 2020. There are many publications on the health impacts of air pollution and some studies have assessed the economic burden of air pollution in India, but there are no studies that have assessed the economic impacts of the different components of air pollution in each state of India.

**Added value of this study**

This study provides the updated estimates of deaths and morbidity attributable to air pollution in every state of India in 2019 based on the improved GBD 2019 methods, which reveal that this burden is higher than was previously estimated. It estimates the economic loss due to lost output from premature death and morbidity attributable to different components of air pollution at the state level based on the updated estimates of deaths and morbidity attributable to air pollution. The findings in this paper highlight that the disease burden attributable to air pollution and its economic impact are high in India, with substantial variations across the states. The wide variations in economic loss attributable to ambient particulate matter pollution, household air pollution, and ambient ozone pollution across the states of India, both in absolute terms and as a percentage of gross domestic product, can be useful for the planning and implementation of targeted interventions at the state level.

**Implications of all the available evidence**

The high burden of air pollution in India and its substantial adverse impact on output could impede India's overall economic development and social wellbeing unless they are addressed as a priority. The variations in these impacts between states indicate that investments in state-specific air pollution control strategies are needed to reduce the significant adverse health and economic impact of air pollution across India.

Diseases attributable to air pollution adversely affect economic growth through reduced productivity and decreased labour supply, and via health-care expenditures and lost welfare.^3^, ^27^, ^28^ In the public health literature, the cost-of-illness method is the main approach used to estimate the economic burden of disease outcomes, including diseases attributable to air pollution.^29^, ^30^, ^31^, ^32^, ^33^, ^34^ The cost-of-illness method includes estimation of direct costs of health care as well as indirect costs, measured as the loss of output due to premature mortality and morbidity.^35^ The output-based approach to estimating indirect cost equates the economic cost of premature mortality to the present value of lost income, and values morbidity by lost output.^33^, ^34^ We use this output-based approach to estimate the economic cost of premature deaths and morbidity attributable to air pollution in each state of India using the GBD 2019 air pollution estimates.

## Methods

### Overview

The India State-Level Disease Burden Initiative estimates disease burden for the states of India as part of GBD. The work of this initiative is approved by the Health Ministry Screening Committee at the Indian Council of Medical Research and the ethics committee of the Public Health Foundation of India. The analysis of the economic impact of air pollution-related diseases and deaths was done on the basis of an invitation extended to the UN Environment Programme by the Indian Ministry of Environment, Forest and Climate Change.

### Estimation of exposure, deaths, and DALYs attributable to air pollution

A detailed description of the GBD methods for estimating deaths and disability-adjusted life-years (DALYs) attributable to air pollution is reported elsewhere,^21^, ^26^ and provided in the appendix (pp 3–14). DALY is a composite metric that combines the years of life lost due to premature death (YLLs) and the years lived with disability (YLDs).

Ambient particulate matter pollution was estimated as exposure to fine particulate matter with an aerodynamic diameter of 2·5 μm or less (PM_2·5_) in a cubic meter of air (μg/m^3^).^21^, ^26^ We used PM_2·5_ as the indicator of ambient particulate matter pollution because it has the strongest association with disease burden and mortality.^36^ Exposure to PM_2·5_ was estimated with use of aerosol optical depth data from multiple satellite sources combined with a chemical transport model and calibration with data from ground-level monitoring station locations in India. Household air pollution was estimated from data on the proportion of individuals using various types of solid fuels for cooking from a number of nationwide surveys. Exposure to solid fuels was converted to PM_2·5_ exposure on the basis of data from a global measurement database including several studies conducted in India.

Ozone exposure was defined as the highest seasonal average 8 h daily maximum concentration, in parts per billion (ppb), with season defined as the 6-month period with the highest mean ozone concentrations. To estimate exposure to ozone in ambient air, ozone ground measurement data from various locations in India were combined with chemical transport models.

Estimates of PM_2·5_ exposure from ambient particulate matter and household air pollution and ambient ozone pollution exposure were used to estimate the attributable burden from various diseases using the standardised GBD comparative risk assessment framework, which uses worldwide literature on the association of each risk factor with particular diseases, as described previously.^26^ We estimated deaths and DALYs attributable to air pollution as a whole and attributable independently to ambient particulate matter, household air pollution, and ambient ozone pollution.

GBD 2019 included a number of methodological updates and new input data for estimation of deaths and DALYs attributable to air pollution, as described elsewhere.^26^ These updates included the addition of burden attributable to ambient particulate air pollution and household air pollution that is mediated by low birthweight and short gestation, along with the previously included attributable burden from chronic obstructive pulmonary disease (COPD), lower respiratory infections, lung cancer, ischaemic heart disease, stroke, type 2 diabetes, and cataract. Another update was the addition of recent studies on air pollution and a new approach to the development of risk curves that enabled exclusion of active smoking studies, which removed an important source of uncertainty related to the differences in exposure between active smoking and air pollution PM_2·5_ sources. A further update was the generation of risk curves for every 5-year age group from 25 years onward for ischaemic heart disease and stroke, which allowed more robust estimates. A detailed description of methodological changes in GBD 2019 and the improvement in estimates from GBD 2017 is available in appendix (pp 3–14) and has been published previously.^26^

We estimated deaths, DALYs, YLLs, and YLDs attributable to air pollution, ambient particulate matter pollution, household air pollution, and ambient ozone pollution for 31 geographical units in India: the 28 states; the union territory of Delhi, the two union territories of Jammu & Kashmir and Ladakh combined, and the other small union territories combined (Andaman and Nicobar Islands, Chandigarh, Dadra and Nagar Haveli, Daman and Diu, Lakshadweep, and Puducherry). We examined the trends in death rates attributable to each of the three components of air pollution from 1990 to 2019. We assessed the Pearson correlation coefficient between the crude DALY rates attributable to each of the three components of air pollution and the per capita gross domestic product (GDP) of the states in 2018–19.^37^ We estimated DALYs and deaths attributable to air pollution in India in 2019 from various diseases.

### Estimation of economic loss attributable to air pollution

We estimated the economic cost of premature mortality by the present discounted value of output lost when a person died in 2019 of pollution-related diseases. The cost of morbidity was estimated as the output lost when a person had pollution-related YLDs in 2019. Both required estimates of output per worker.

The output per worker in a given state in 2019 was calculated as the labour share of GDP multiplied by GDP in 2018–19,^37^ divided by the number of people who were employed. The labour share of GDP in each state was estimated using data from the Penn World Tables 21.^38^ Workers of all ages in a state were assumed to produce the same output per worker. Because not all people of a given age were working, output per worker was adjusted by the fraction of people in each age group who were working. This information was obtained from the National Sample Survey on employment and unemployment for 2011–12.^39^ To predict output in future years, output per worker was assumed to grow at the historical real rate of growth of output per worker, estimated using data from the KLEMS database.^40^ For people not working, expected output per worker in each year was assumed to be equal to 30% of market output to allow for non-market production.^41^

To quantify the output losses in future years if a person of a given age dies in the current year requires estimating the present discounted value of their future output. An individual's output at each age is the product of output per worker (as described above) and the probability that a person is working at each age, measured as the ratio of the working population to the total population at that age. This estimate of lost output must be adjusted to reflect the probability a person survives to each future age. Survival probabilities were estimated using state-specific life tables from GBD 2019. Expected future output at each age was discounted to the present at a rate of interest of 6%, taken to be the yield on 10-year Indian Government bonds in late 2020. A sensitivity analysis was done to examine the impact of using different discount rates between 4% and 8% on the estimate of economic loss.

The total output lost through premature mortality attributable to air pollution was estimated as the present discounted value of lost market and non-market output for a person who dies in 2019 at each age multiplied by the number of deaths due to air pollution in 2019 for that age, with the result summed over all ages. The present value of lost output per person over the remainder of the person's working life is a conservative estimate of the loss in output that is a consequence of premature death.

To estimate the total output losses attributable to air pollution-related morbidity, the expected market and non-market output loss per person in 2019, by age group, was multiplied by the YLDs attributable to air pollution in 2019 for each age group and the result summed over all ages.^21^ Details of these methods are presented in the appendix (pp 15–17). We report the output losses in monetary terms and as a percentage of GDP for deaths and YLDs attributable to air pollution, and separately for ambient particulate matter pollution, household air pollution, and ambient ozone pollution, for every state of India in 2019 and for India overall by aggregating the state estimates.

### Role of the funding source

Some of the contributors to this study work with the Indian Council of Medical Research and the UN Environment Programme. The other funder, the Bill & Melinda Gates Foundation, had no role in the study design, data collection, data analysis, data interpretation, or writing of the report. The corresponding author had full access to all the data in the study and had final responsibility for the decision to submit for publication. All authors had access to the estimates presented in the paper.

## Results

**Figure 1:**
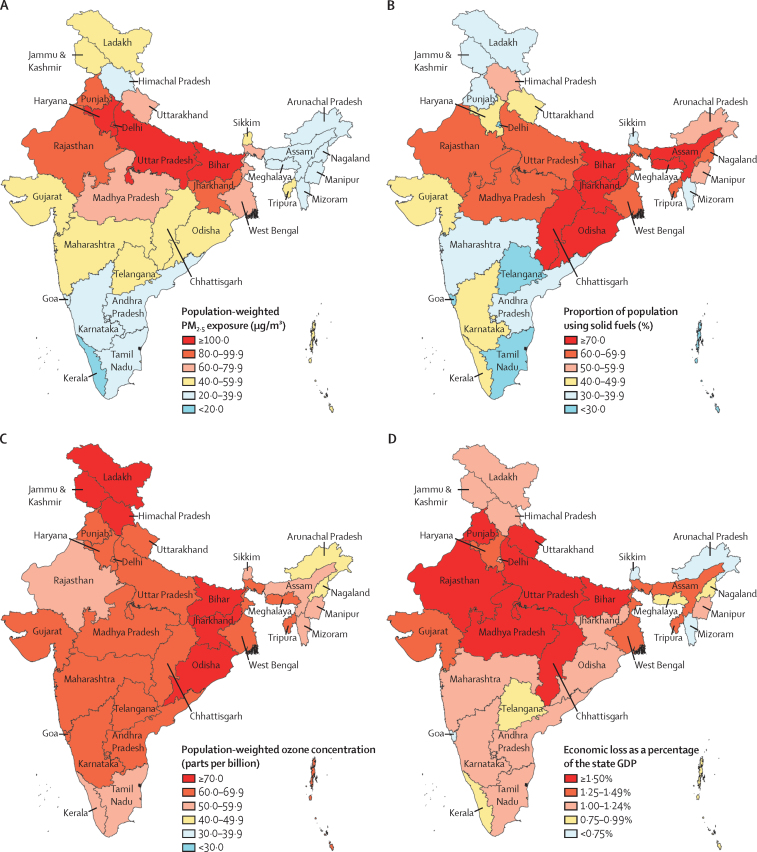
Exposure to air pollution and economic loss due to premature deaths and morbidity attributable to air pollution in the states of India, 2019 (A) Population-weighted mean ambient PM_2·5_ concentration. (B) Proportion of population using solid fuels. (C) Population-weighted ozone concentration in parts per billion. (D) Economic loss due to premature deaths and morbidity attributable to air pollution as a percentage of the state GDP. GDP=gross domestic product. PM_2·5_=fine particulate matter with an aerodynamic diameter of 2·5 μm or less.

The annual average population-weighted mean PM_2·5_ concentration (as a measure of ambient particulate matter exposure) was 91·7 μg/m^3^ (95% uncertainty interval [UI] 69·6–113·9) in India in 2019. Across the states of India, exposure to ambient particulate matter ranged from 15·8 μg/m^3^ (13·0–18·7), in Kerala, to 217·6 μg/m^3^ (117·9–297·3), in Delhi—a 13·8 times difference. Higher concentrations were found in the northern states, including the four states with the highest exposures (123·5–217·6 μg/m^3^; figure 1A; appendix p 18). The proportion of the population using solid fuels for cooking in India in 2019 was 56·3% (55·1–57·4). This proportion was highest in the eastern and northern states, with proportion above 70% in six states (figure 1B; appendix p 18). Use of solid fuels contributed an average 82·8 μg/m^3^ PM_2·5_ (41·9–153·8) in households, in addition to the ambient 91·7 μg/m^3^ PM_2·5_ present in India in 2019 (appendix p 18). The average ambient ozone concentration in India in 2019 was 66·2 ppb (66·0–66·3), ranging from 47·4 ppb (46·3–48·5), in Arunachal Pradesh, to 76·6 ppb (75·8–77·4), in Jammu & Kashmir and Ladakh (figure 1C; appendix p 18).

**Figure 2:**
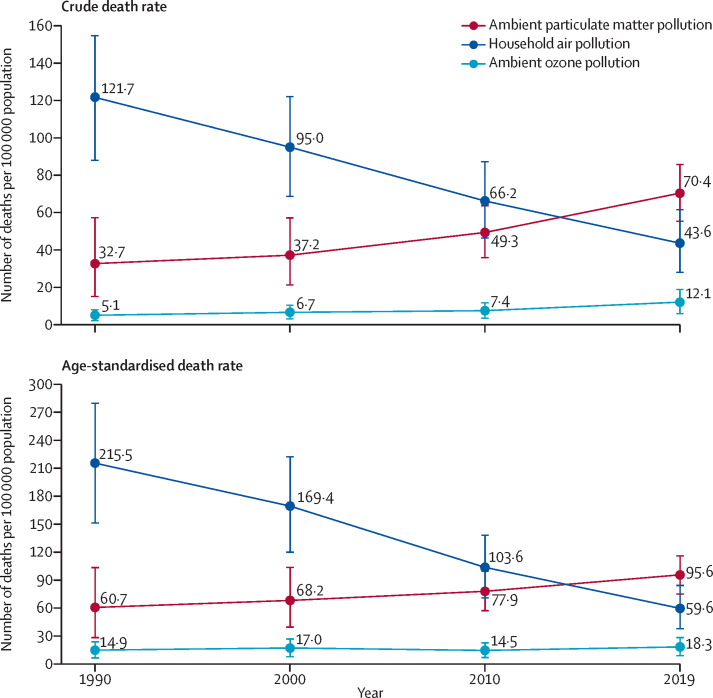
Death rate attributable to ambient particulate matter pollution, household air pollution, and ambient ozone pollution per 100 000 population in India, 1990–2019

In 2019, 1·67 million (95% UI 1·42–1·92) deaths in India were attributable to air pollution, accounting for 17·8% (15·8–19·5) of the total deaths in India (table 1).^42^ 0·98 million (0·77–1·19) deaths were attributable to ambient particulate matter pollution, 0·61 million (0·39–0·86) to household air pollution, and 0·17 million (0·08–0·26) to ambient ozone pollution (table 1). The crude death rate per 100 000 population due to household air pollution decreased in India by 64·2% (52·2–74·2) from 1990 to 2019, while that due to ambient particulate matter pollution increased by 115·3% (28·3–344·4) and that due to ambient ozone pollution increased by 139·2% (96·5–195·8; figure 2; appendix p 19). The age-standardised death rate due to household air pollution decreased by 72·3% (63·6–79·8) from 1990 to 2019 and that due to ambient ozone pollution increased by 23·2% (0·5–52·0), while the 95% UI of the estimated 57·4% increase in death rate due to ambient particulate matter pollution overlapped with zero (–4·4 to 225·3; figure 2).

**Table 1:** Deaths and DALYs attributable to air pollution in India in 2019

	Number of deaths, millions*	**Percentage of total deaths**†	Number of DALYs, millions*	**Percentage of total DALYs**†
Air pollution	1·67 (1·42–1·92)	17·8% (15·8–19·5)	53·5 (46·6–60·9)	11·5% (10·2–12·8)
Ambient particulate matter pollution	0·98 (0·77–1·19)	10·4% (8·4–12·3)	31·1 (24·6–37·5)	6·7% (5·3–8·0)
Household air pollution	0·61 (0·39–0·86)	6·5% (4·3–9·0)	20·9 (14·1–28·7)	4·5% (3·0–6·1)
Ambient ozone pollution	0·17 (0·08–0·26)	1·8% (0·9–2·7)	3·06 (1·51–4·83)	0·7% (0·3–1·0)

Data are point estimate (95% UI). DALYs=disability-adjusted life-years.

* The sums of deaths and DALYs attributable to each component of air pollution are more than the estimates for overall air pollution because the population attributable fractions from component risk factors can add up to more than the population attributable fraction for the parent risk factor, even if the components are independent.

† In 2019, 9·39 million total deaths and 467·8 million total DALYs were estimated for India.^42^

11·5% of the total DALYs in India in 2019 were attributable to air pollution,^42^ the majority of which were due to ambient particulate matter pollution (6·7% [5·3–8·0]) and household air pollution (4·5% [3·0–6·1]; table 1). The crude DALY rate attributable to ambient particulate matter pollution varied 5·5 times across the states in 2019, with several northern states having the highest rates (appendix p 20). The crude DALY rate attributable to household air pollution varied 132·3 times, with the highest rates in the northern and northeastern states (appendix p 20). The crude DALY rate attributable to ambient ozone pollution varied 11·2 times across the states in 2019, with a mixed pattern with regard to geographical location (appendix p 20). The crude DALY rate attributable to household air pollution had a significant inverse correlation with the per-capita GDP of the states (r=–0·71, r^2^=0·50; p<0·0001), but there was no significant correlation between the per-capita GDP of the states and the crude DALY rate attributable to ambient particulate matter pollution (r=0·25, r^2^=0·06, p=0·17) or ambient ozone pollution (r=–0·11, r^2^=0·01, p=0·56).

**Figure 3:**
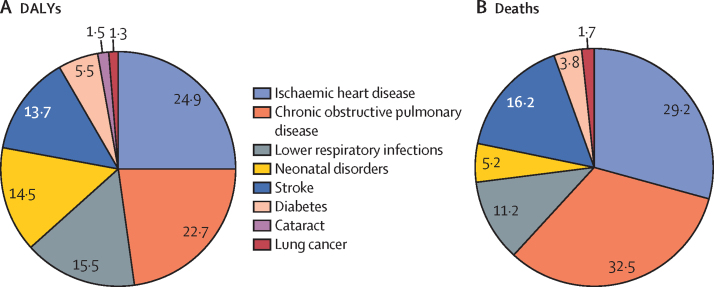
Causes of DALYs (A) and deaths (B) attributable to air pollution in India, 2019 Individual causes are shown as a percentage of total DALYs or deaths. DALYs=disability-adjusted life-years.

Of the total DALYs attributable to air pollution in India in 2019, 39·5% were from lung diseases, which included COPD (22·7%), lower respiratory infections (15·5%), and lung cancer (1·3%; figure 3A). The remaining DALYs were from ischaemic heart disease (24·9%), stroke (13·7%), diabetes (5·5%), neonatal disorders (14·5%), and cataract (1·5%). Of the total deaths attributable to air pollution in India in 2019, the largest proportions were due to COPD (32·5%) and ischaemic heart disease (29·2%), followed by stroke (16·2%) and lower respiratory infections (11·2%; figure 3B).

The economic loss due to lost output from premature deaths attributable to air pollution in India in 2019 was US$28·8 billion (95% UI 21·4–37·4), and from morbidity attributable to air pollution was $8·0 billion (5·9–10·3; table 2; appendix p 21). Of the total economic loss of $36·8 billion (27·4–47·7) attributable to air pollution in India in 2019, 36·6% was from lung diseases, which included COPD (21·1%), lower respiratory infections (14·2%), and lung cancer (1·2%), and the rest was from ischaemic heart disease (24·9%), stroke (14·1%), diabetes (8·4%), neonatal disorders (13·3%), and cataract (2·7%).

**Table 2:** Total and per-capita economic loss due to premature deaths and morbidity attributable to air pollution in the states of India, 2019

	**Premature deaths, US$ millions**	**Morbidity, US$ millions**	**Total, US$ millions**	**Per capita, US$**
India	28 799 (21 429–37 421)	8005 (5940–10 289)	36 804 (27 369–47 710)	26·5 (19·7–34·3)
Bihar	1257 (931–1646)	296 (223–376)	1553 (1153–2022)	12·7 (9·4–16·6)
Uttar Pradesh	4255 (3153–5508)	876 (663–1108)	5130 (3816–6616)	21·1 (15·7–27·2)
Manipur	30 (21–41)	11 (8–14)	40 (29–55)	11·5 (8·2–15·6)
Jharkhand	408 (297–542)	136 (101–173)	543 (398–715)	14·3 (10·5–18·9)
Madhya Pradesh	1614 (1212–2090)	356 (268–452)	1970 (1480–2542)	22·2 (16·7–28·7)
Assam	528 (389–698)	129 (95–167)	657 (483–865)	18·2 (13·4–24·0)
Meghalaya	30 (20–43)	9 (7–12)	39 (27–55)	11·5 (7·9–16·0)
Jammu & Kashmir and Ladakh	201 (150–261)	51 (38–66)	252 (188–327)	18·0 (13·4–23·3)
Chhattisgarh	549 (409–712)	141 (103–182)	690 (512–894)	21·8 (16·1–28·2)
West Bengal	1607 (1233–2018)	519 (389–659)	2125 (1623–2677)	21·3 (16·3–26·9)
Nagaland	26 (17–37)	8 (6–10)	34 (23–47)	17·2 (11·8–24·0)
Odisha	609 (430–831)	197 (144–257)	807 (574–1088)	17·3 (12·3–23·3)
Rajasthan	1902 (1376–2504)	392 (298–492)	2294 (1674–2996)	28·5 (20·8–37·3)
Tripura	70 (50–94)	21 (16–27)	91 (66–121)	22·6 (16·4–30·1)
Arunachal Pradesh	19 (13–28)	7 (5–9)	26 (18–37)	15·1 (10·2–21·2)
Mizoram	17 (11–24)	6 (4–7)	22 (15–31)	17·6 (12·0–24·5)
Andhra Pradesh	1007 (717–1373)	342 (252–445)	1349 (969–1818)	24·9 (17·9–33·5)
Punjab	920 (695–1176)	229 (167–298)	1149 (862–1474)	37·0 (27·7–47·4)
Tamil Nadu	1886 (1397–2457)	643 (460–853)	2529 (1857–3310)	31·7 (23·3–41·5)
Maharashtra	3003 (2279–3835)	972 (725–1245)	3975 (3004–5080)	31·9 (24·1–40·7)
Telangana	841 (591–1153)	275 (202–356)	1116 (793–1508)	28·7 (20·4–38·7)
Kerala	741 (555–962)	349 (253–458)	1091 (808–1421)	31·2 (23·1–40·6)
Himachal Pradesh	192 (142–251)	62 (46–80)	254 (188–331)	33·3 (24·7–43·5)
Karnataka	2113 (1593–2718)	568 (413–741)	2681 (2006–3459)	39·4 (29·5–50·9)
Uttarakhand	413 (309–537)	114 (84–146)	527 (393–683)	44·5 (33·2–57·7)
Gujarat	2288 (1728–2938)	571 (429–729)	2860 (2158–3667)	41·3 (31·2–53·0)
Haryana	1224 (929–1575)	342 (259–434)	1566 (1188–2009)	53·8 (40·8–69·0)
Other small union territories	86 (60–118)	35 (25–46)	120 (85–164)	31·7 (22·5–43·2)
Sikkim	17 (12–24)	8 (6–11)	25 (18–35)	38·6 (27·3–52·5)
Delhi	893 (672–1153)	314 (235–402)	1207 (906–1555)	62·0 (46·6–79·9)
Goa	54 (37–75)	26 (19–35)	80 (56–110)	52·2 (36·3–71·9)

The states are listed in increasing order of per-capita gross domestic product in 2018–19.

The economic loss due to lost output from premature deaths and morbidity attributable to air pollution was 1·36% (95% UI 1·01–1·76) of India's GDP in 2019 (appendix p 22). A sensitivity analysis showed that, with a discount rate of 4% instead of 6%, the economic loss would be 1·86% of the GDP, and with a discount rate of 8% it would be 1·10% of the GDP (appendix p 15). The economic loss attributable to air pollution as a percentage of state GDP varied from 0·67% (0·47–0·91) to 2·15% (1·60–2·77)—a 3·2 times difference—across the states, and was highest in the states of Uttar Pradesh (2·15%), Bihar (1·95%), Madhya Pradesh (1·70%), Rajasthan (1·70%), and Chhattisgarh (1·55%), which have a relatively low per-capita GDP, and in Punjab (1·52%) and Uttarakhand (1·50%) which have relatively high per-capita GDP (figure 1D; appendix p 22). The per-capita economic loss due to air pollution in India was $26·5 (19·7–34·3), and varied 5·4 times across the states; this economic loss per capita was highest in Delhi ($62·0 [46·6–79·9]) and Haryana ($53·8 [40·8–69·0]) and was generally higher in the states with high per-capita GDP (table 2).

**Figure 4:**
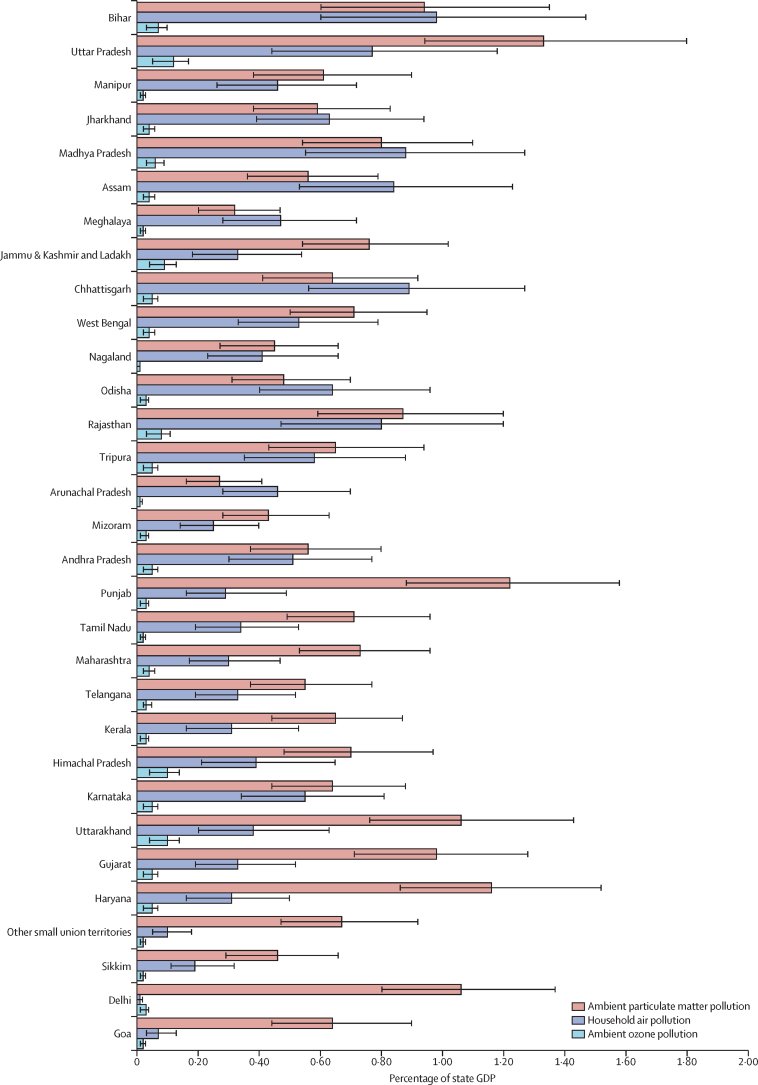
Economic loss due to premature deaths and morbidity attributable to ambient particulate matter pollution, household air pollution, and ambient ozone pollution as a percentage of state GDP in India, 2019 States are listed in increasing order of per-capita GDP in 2018–19. Error bars represent 95% uncertainty intervals. GDP=gross domestic product.

The economic loss due to lost output from premature deaths and morbidity attributable to ambient particulate matter pollution as a percentage of GDP in India was 0·84% (0·59–1·13) in 2019 (appendix p 22). This proportion varied 4·9 times across the states (from 0·27% [0·16–0·41] to 1·34% [0·94–1·80]) and was highest in Uttar Pradesh (1·34%) which has a relatively low per-capita GDP, followed by Punjab (1·22%), Haryana (1·16%), Uttarakhand (1·06%), and Delhi (1·06%), which have a relatively high per-capita GDP (figure 4; appendix p 22).

The economic loss due to lost output from premature deaths and morbidity attributable to household air pollution as a percentage of state GDP in India was 0·49% (0·29–0·75) in 2019, with 110·3 times variation (from 0·01% [0·00–0·02] to 0·98% [0·60–1·47]) across the states (figure 4; appendix p 22). This proportion was highest in Bihar (0·98%), Chhattisgarh (0·89%), Madhya Pradesh (0·88%), Assam (0·84%), Rajasthan (0·79%), and Uttar Pradesh (0·77%), which have a relatively low per-capita GDP.

In 2019, the economic loss due to lost output from premature deaths attributable to ambient ozone pollution as a percentage of GDP in India was 0·05% (0·02–0·09) and varied 11·2 times across the states, ranging from 0·01% (0·00–0·02) in Nagaland to 0·12% (0·05–0·20) in Uttar Pradesh (figure 4; appendix p 22).

The economic loss due to lost output from premature deaths and morbidity attributable to ambient particulate matter pollution ranged from $9·5 million in the small northeastern state of Arunachal Pradesh to $3188·4 million in the northern state of Uttar Pradesh, and that attributable to household air pollution ranged from $7·6 million in the small western state of Goa to $1829·6 million in Uttar Pradesh (appendix p 21). The economic loss due to lost output from premature deaths attributable to ambient ozone pollution ranged from $0·4 million in the small northeastern state of Nagaland to $286·2 million in Uttar Pradesh (appendix p 21).

## Discussion

Important revisions in the GBD 2019 methods have led to more robust estimates of deaths and DALYs attributable to air pollution compared with the previous GBD estimates. The main contributors to the higher estimates in GBD 2019 are the inclusion of disease burden attributable to air pollution mediated by low birthweight and short gestation, and updated relative risk curves, particularly for stroke, with the availability of recent evidence, including from India.^26^, ^43^, ^44^, ^45^ These method updates resulted in an increased estimate of the burden attributable to air pollution in India, which accounted for an estimated 1·67 million deaths in India in 2019. For comparison with the estimate of 1·24 million (1·09–1·39) air pollution attributable deaths in India in 2017 in GBD 2017,^21^ the 2017 estimate for India in GBD 2019 is 1·60 million (1·41–1·80) deaths.

The burden of household air pollution decreased substantially in India between 1990 and 2019; however, the burden attributable to ambient particulate matter pollution and ambient ozone pollution increased during this period. In 2019, the less developed states in north and northeastern India had a higher burden from household air pollution than the more developed states, whereas states in northern India had a high burden of ambient particulate matter pollution irrespective of whether they were less or more developed.

The economic loss due to lost output from premature deaths and morbidity attributable to air pollution is high in India, equivalent to 1·36% of India's GDP in 2019. A further source of economic loss is the health-care cost of treating diseases attributable to air pollution. Based on National Health Accounts data,^46^ we estimated the total health-care cost in India in 2019 to be $103·7 billion. With air pollution responsible for 11·5% of the disease burden (measured as DALYs) in India in 2019, a crude estimate of the health-care cost for air pollution-related diseases would be $11·9 billion (or 0·44% of India's GDP).

In 2019, there was a three-fold variation between the states with regard to the economic loss due to lost output from premature deaths and morbidity attributable to air pollution as a percentage of state GDP, and a five-fold variation in absolute per-capita economic loss, with a relatively higher burden in the northern states compared with the other states of India. The economic loss due to premature mortality and morbidity is a disinvestment in human capital stock.^33^ Human capital is a broad concept, defined as the stock of knowledge and skills possessed by a population and the health status of that population, which is an important component of the inclusive wealth of a nation.^47^, ^48^, ^49^

The increasing death rate attributable to ambient particulate matter pollution reflects increasing pollutant emissions from rising energy consumption, accelerated urbanisation, rapid industrialisation, and growing numbers of petroleum-powered vehicles.^50^ Evidence suggests that climate change can amplify the adverse impacts of air pollution through atmospheric stagnation, temperature-driven increases in PM_2·5_ concentration, and ground-level ozone formation, which are likely to be particularly severe in India.^51^, ^52^ The economic burden due to lost productivity will increase in magnitude in the years ahead if air pollution continues to worsen. If air pollution is not aggressively controlled and managed, its great costs could not only undermine plans to increase India's economy to $5·0 trillion by 2024, but would also impede the growth in inclusive wealth of the nation through reduced human capital stock.

The total health expenditure in India is 3·8% of GDP,^46^ while the economic loss due to lost output from premature deaths and morbidity attributable to air pollution estimated in this study was 1·36% of GDP, indicating that the total economic impact of air pollution is high. The loss of output in monetary terms attributable to air pollution at the state level is associated with the number and the age-distribution of deaths and morbidity in each state and state GDP per worker. The economic loss due to air pollution as a percentage of state GDP was highest in northern states of India because people in these states are exposed to very high concentrations of ambient PM_2·5_ and a high proportion of their population uses solid fuels. The states of Uttar Pradesh and Bihar, with the highest economic loss as a percentage of their GDP, had the lowest per-capita GDP among the states of India, indicating that these poor states are most vulnerable to the adverse economic impacts of air pollution.

Several studies have evaluated the economic impacts of premature mortality and morbidity attributable to air pollution, in India and globally, using various approaches. A study using the output-based approach and GBD 2013 mortality data estimated the total forgone labour output due to air pollution in India in 2013 to be 0·84% of GDP.^33^ This estimate is lower than our estimate of 1·36% of GDP because the former provided estimates for output lost due to premature mortality only and was based on earlier estimates of PM_2·5_ mortality. This study also estimated the loss of economic welfare due to premature mortality attributable to air pollution, using the willingness-to-pay approach, to be 7·7% of GDP in India in 2013.^33^ The estimated loss of economic welfare is much higher than the estimates of productivity loss alone because what people are willing to pay to reduce their risk of death from a risk factor or disease is generally always higher than the present value of lost output.^3^ Moreover, the willingness-to-pay approach values all premature mortality attributable to air pollution, whereas the output-based approach considers premature mortality only in working-age groups.^3^ The willingness-to-pay approach is also sensitive to the assumptions associated with the estimation of the value per statistical life, which has wide variations in the studies reported from India.^53^

In other countries, the human capital-augmented production function framework for estimating the macroeconomic cost of air pollution in China resulted in an annual economic loss accounting for 0·5% of GDP.^54^ Other studies used a damage function approach and estimated the morbidity and mortality effects of particulate matter pollution on the population as 3·4% of GDP in 1999 in Singapore^32^ and 1% of GDP in Jakarta during the same period.^55^ These variations in the magnitude of economic burden attributable to air pollution across different studies in India and other countries are likely to be due to geographical differences in the patterns of premature deaths and morbidity, as well as differences in labour force dynamics, types of data used, and methodological approaches.

Studies on sources of emission in major cities of India have identified a number of important contributing sources, although there is variation over space and time, and uncertainty in their contributions because of the use of different methods and underlying uncertainty in source signatures and emission inventories.^11^, ^12^, ^13^, ^14^, ^16^, ^56^ These studies have highlighted the contributions of industrial sources, energy production, and especially residential emissions from the use of polluting fuels for cooking and heating. In urban areas, contributions from transportation sources are also important.^12^, ^14^, ^16^

The Government of India has developed a series of programmes to monitor and control ambient air pollution (appendix pp 23–26). The National Air Quality Monitoring Programme was initiated in 1984 to determine the status and trends of ambient air quality, which now extends to 339 cities in 29 Indian states or union territories and operates 779 air quality monitoring stations.^57^, ^58^, ^59^, ^60^ In 2019, the National Clean Air Programme was launched, which coordinates air pollution control efforts across sectors, educates the Indian public about the importance of clean air for health, and aims for 20–30% reductions in PM_2·5_ and PM_10_ concentrations by 2024 in 102 cities.^60^, ^61^ The Smart City Mission was launched in 2015 to develop 100 citizen-friendly and sustainable cities across the country.^62^ Based on the severity of ambient air pollution, these programmes along with others are tailored to the local situation in each city.

Evidence suggests that household air pollution in India contributes substantially to ambient particulate matter pollution.^63^ Therefore, the programmes aimed at controlling household air pollution have a double benefit by also reducing ambient particulate matter. Several attempts have been made to reduce the household air pollution in India through various government programmes,^64^, ^65^, ^66^ including Unnat Chulha Abhiyan, launched in 2014 to provide modified biomass cook stoves to low-income households, and the Pradhan Mantri Ujjwala Yojana programme, launched in 2016 to provide liquefied petroleum gas to 80 million low-income households. The Pradhan Mantri Ujjwala Yojana programme has been highly successful and has exceeded its target in 2019.^67^, ^68^, ^69^, ^70^, ^71^ However, additional efforts are required to achieve consistent usage of liquefied petroleum gas for cooking. The full realisation of the social, economic, and health benefits of household air pollution reduction can be achieved by overcoming the continuing challenges of limited translation of initial liquefied petroleum gas adoption to sustained adoption and limited abandonment of traditional fuels.^72^ This could be achieved by implementation research that explores the social, economic, and cultural factors influencing clean fuel adoption.^73^

The improvements in air quality across India during the COVID-19 lockdown period,^74^ and its upsurge again with the easing of restrictions,^75^ provide interesting pointers to the extent of air pollution reduction that is possible with reduced human activity. Evidence also suggests that exposure to air pollution is associated with increased risk of morbidity and mortality from COVID-19.^76^, ^77^ Therefore, reduction in air pollution could help in reducing the adverse effect of COVID-19 as well.

Air pollution has the potential to impede accumulation of future human capital by reducing children's survival, undermining their health, and reducing their ability to benefit from education.^78^ The cost savings resulting from the prevention of productivity losses attributable to air pollution would contribute to the formation of new human capital. The potential magnitude of the benefits, both for human health and the economy, of investing in air pollution control strategies can be seen in the experience of the USA, where every dollar invested in the control of ambient air pollution since 1970 is estimated to have yielded an economic benefit of $30, based on the willingness-to-pay approach.^3^ There has been a substantial reduction in air pollution in the USA over the past few decades along with significant economic growth,^79^ indicating that the successful implementation of air pollution control strategies could help in improving the health of the population, even when the economy is growing. The reduction of airborne lead pollution through removal of lead from gasoline in the USA has also been linked with boosted economic output through reductions in children's blood lead concentrations, thereby increasing their intelligence, creativity, and economic productivity.^80^ These findings indicate that investing in control of air pollution in India could be highly cost-effective and pay for itself many times over.

There are several limitations of this study. First, our estimates of premature deaths and morbidity attributable to pollution are conservative because they are based on air pollution–disease pairs for which the evidence of causality is considered adequate in the GBD analysis.^21^, ^26^ Air pollution could potentially lead to other adverse outcomes as well, such as dementia^81^ and loss of intelligence quotient,^82^ but conclusive evidence for such associations is not yet available. Additionally, the disease burden attributable to air pollution in GBD is limited to that related to long-term exposure to ambient PM_2·5_, household air pollution, and ozone, and does not yet consider additional pollutants such as nitrogen dioxide or the impacts of short-term variations in exposure.^83^ Furthermore, GBD estimates of household air pollution include only solid fuels used for cooking and not for heating. Second, we have not quantified the direct health-care costs and other potentially negative economic impacts of air pollution, such as effects on tourism or ecosystem services. Third, our output estimates depend on a number of assumptions, which, if changed, would alter the estimates. For simplicity, we assumed that the rate of growth in real output per worker is the same for all the states and that labour's share of GDP remains constant at its current value. The state-specific life tables were assumed to remain constant over the lifetimes of people currently alive, which is likely to understate the economic losses in less developed states, where survival probabilities are likely to increase in the future. Our results are also dependent on the rate at which future output is discounted. Even with these limitations, our study provides useful estimates of economic loss attributable to air pollution in every state of India using the most recent air pollution burden data.

The findings in this report should motivate the central and state governments to allocate sufficient long-term funding to prevent the adverse health impacts of air pollution. Control of air pollution in India will not only improve health as envisioned in the SDGs, but will also accelerate the potential to achieve other SDG targets, including alleviating poverty, promoting social justice, enhancing the liveability of India's cities, and reducing the pace of climatic changes. Air pollution control in India is not an expenditure, but rather an essential investment in the country's future economic growth. Strengthening the ongoing efforts to manage and prevent air pollution would help in avoiding the substantial economic losses attributable to air pollution in the states of India.

## Data sharing

The air pollution exposure and disease burden data used in these analyses are available at http://ghdx.healthdata.org/gbd-2019, https://vizhub.healthdata.org/gbd-compare/india, and from the authors on request. The economic data used in these analyses are available from the authors on request.
