# Supplementary material for: Health and economic impact of air pollution in the states of India: the Global Burden of Disease Study 2019
Source: Lancet Planet Health. 2020 Dec 27;5(1):e25–38. doi: 10.1016/S2542-5196(20)30298-9 (PMC7805008; doi:10.1016/S2542-5196(20)30298-9)
Supplement: Supplementary appendix [file mmc1.pdf]

THE LANCET
Planetary Health

Supplementary appendix

This appendix formed part of the original submission and has been peer reviewed. We post it as supplied by the authors.

Supplement to: India State-Level Disease Burden Initiative Air Pollution Collaborators. Health and economic impact of air pollution in the states of India: the Global Burden of Disease Study 2019. *Lancet Planet Health* 2020; published online Dec 22. http://dx.doi.org/10.1016/S2542-5196(20)30298-9.

Health and economic impact of air pollution in the states of India: the Global Burden of Disease Study 2019

India State-Level Disease Burden Initiative Air Pollution Collaborators

Web Appendix

Correspondence to: Prof. Lalit Dandona, lalit.dandona@icmr.gov.in

Table of contents

1. GBD 2019 air pollution estimation methods....3
2. Estimation of output losses due to air pollution....15
3. $PM_{2.5}$ concentration, use of solid fuels, and ozone concentration in the states of India, 2019....18
4. Deaths attributable to air pollution in the states of India, 2019....19
5. YLL, YLD, and DALY rates attributable to air pollution in the states of India, 2019....20
6. Economic loss due to premature deaths and morbidity attributable to air pollution in the states of India, 2019 21
7. Economic loss due to premature deaths and morbidity attributable to air pollution as a percentage of state GDP in India, 2019....22
8. Air pollution control initiatives in India....23

1. GBD 2019 air pollution estimation methods

The materials presented here are reproduced or adapted from:

GBD 2019 Risk Factors Collaborators. Global burden of 87 risk factors in 204 countries and territories, 1990–2019: a systematic analysis for the Global Burden of Disease Study 2019 *Lancet* 2020; 396: 1223–49.

GBD complies with the Guidelines for Accurate and Transparent Health Estimates Reporting (GATHER) recommendations.

The components of air pollution risk factor, main model types used, and the main data sources for exposure in GBD 2019 are summarised below:

Risk factor	Level	Model type	Main data source for exposure
**Air pollution**	**2**		
Particulate matter pollution	3		
Ambient particulate matter pollution	4	Bayesian hierarchical model of grid-level fusion of satellite-based estimates, chemical transport models and ground level monitoring data	Atmospheric chemical transport models, satellite measurements of aerosols in the atmosphere, data from ground-level monitoring sites
Household air pollution from solid fuels	4	Spatio-Temporal-Gaussian Process Regression (ST-GPR)	Population surveys and censuses; exposure mapping model
Ambient ozone pollution	3	Ensemble of multiple chemical transport model estimates that is bias-corrected with ground measurements in a geospastistical framework	Atmospheric chemical transport models; data from ground-level monitoring sites

A comprehensive description of the metrics, data sources, and statistical modelling for GBD 2019 has been reported elsewhere.[1] The GBD methods relevant for air pollution estimation in India are described in detail elsewhere.[2] Here we present a brief summary of the data and estimation methods highlighting the major updates in GBD 2019.

A. Ambient particulate matter pollution

Exposure to ambient particulate matter pollution is defined as the population-weighted annual average mass concentration of particles with an aerodynamic diameter less than 2.5 micrometers ($PM_{2.5}$) in a cubic meter of air. This measurement is reported in µg/m3. These estimates were based on multiple satellite observations of aerosols in the atmosphere, ground measurements, chemical transport model simulations, population estimates, and land-use data.

Data

The estimates of ambient $PM_{2.5}$ exposures in India were based on multiple satellite-based aerosol optical depth data combined with a chemical transport model, and calibration of these with $PM_{2.5}$ data from ground-level monitoring stations.

Ground measurements used for GBD 2019 include updated measurements from sites included in GBD 2017 and additional measurements from new locations. The data include measurements of concentrations of PM_{10} and $PM_{2.5}$ from 10,408 ground monitors from 116 countries from 2010 to 2017. For locations measuring only PM_{10}, $PM_{2.5}$ measurements were estimated from PM_{10} using a hierarchy of conversion factors ($PM_{2.5}/PM_{10}$ ratios). Estimates in GBD 2019 included a substantially increased number of ground monitoring sites from India, including data from 185 ground monitors for $PM_{2.5}$ and 184 monitors for PM_{10}.

Satellite-based estimates

The global geophysical $PM_{2.5}$ estimates for the years 2000–2017 are from Hammer and colleagues Version V4.GL.03.NoGWR used at 0.1°x0.1° resolution (~11 x 11 km resolution at the equator) and described in details elsewhere.[1] The method is based on the algorithms of van Donkelaar and colleagues (2016) as used

in GBD 2017,[3] with updated satellite retrievals, chemical transport modelling, and ground-based monitoring. The algorithm uses aerosol optical depth (AOD) from several updated satellite products (MAIAC, MODIS C6.1, and MISR v23), including finer resolution, increased global coverage, and improved long-term stability. Ground-based observations from a global sunphotometer network (AERONET version 3) were used to combine different AOD information sources. This is the first time that data from MAIAC at 1 km resolution was used to estimate $PM_{2.5}$ at the global scale. The GEOS-Chem chemical transport model with updated algorithms was used for geophysical relationships between surface $PM_{2.5}$ and AOD. Updates to the GEOS-Chem simulation included improved representation of mineral dust and secondary organic aerosol, as well as updated emission inventories. The resultant geophysical $PM_{2.5}$ estimates are highly consistent with ground monitors worldwide (R^2=0.81, slope = 1.03, n = 2541).

Population data

A comprehensive set of population data, adjusted to match UN2015 Population Prospectus, on a high-resolution grid was obtained from the Gridded Population of the World (GPW) database. Estimates for 2000, 2005, 2010, 2015, and 2020 were available from GPW version 4, with estimates for 1990 and 1995 obtained from the GPW version 3. These data were provided on a 0.0083o× 0.0083o resolution. Aggregation to each 0.1o×0.1o grid cell was accomplished by summing the central 12 × 12 population cells. Populations estimates for 2001–2004, 2006–2009, 2011–2014 and 2016–2019 were obtained by interpolation using natural splines with knots placed at 2000, 2005, 2010, 2015, and 2020. This was performed for each grid cell.

Chemical transport model simulations

Estimates of the sum of particulate sulphate, nitrate, ammonium and organic carbon and the compositional concentrations of mineral dust simulated using the GEOS Chem chemical transport model, and a measure combining elevation and the distance to the nearest urban land surface were available from 2000 to 2017 for each 0.1×0.1o grid cell.[3]

Modelling strategy

The Data Integration Model for Air Quality (DIMAQ2) was used for ambient particulate matter pollution modelling in GBD 2019. Due to the complexity of the models, the size of the data, and the number of spatial predictions required, an “approximate” Bayesian inference based on integrated nested Laplace approximations (INLA) were performed[4] using the R interface to the INLA computational engine (R-INLA). GBD 2019 also makes use of an innovation in the way that samples from the (Bayesian) model were used to represent distributions of estimated concentrations in each grid-cell. Here estimates, and distributions representing uncertainty, of concentrations for each grid are obtained by taking repeated (joint) samples from the posterior distributions of the parameters and calculating estimates based on a linear combination of those samples and the input variables.[5]

DIMAQ2 was used to produce estimates of ambient $PM_{2.5}$ for 1990, 1995, and 2010–2019 by matching the gridded estimates with the corresponding coefficients from the calibration. As there is a lag in reporting ambient air pollution based quantities, the input variables were extrapolated allowing estimates for 2018 and 2019 to be produced in the same way as other years and, crucially, allowing measures of uncertainty to be produced within the Bayesian Hierarchical Model framework rather than by using post-hoc approximations.

Estimates from the satellites and the GEOS-Chem chemical transport model in 2018 and 2019 were produced by extrapolating estimates from 2000–2017 using generalised additive models,[6] on a cell by-cell basis, except in those grid cells that saw a >100% increase between 2016 and 2017, in which case only the 2000–2016 estimates were used for extrapolating, in order to avoid unrealistic and/or unjustified extrapolation of trends. Population estimates for 2018 and 2019 were obtained by interpolation as described above.

All modelling was performed on the log-scale. The choice of which variables were included in the model was made based on their contribution to model fit and predictive ability. The following is a list of variables and model structures that were considered in developing the GBD 2019 model:

Variable	Model structure
Continuous explanatory variables	(SAT) Estimate of $PM_{2.5}$ (in μgm-3) from satellite remote sensing on the log scale.
	(POP) Estimate of population for the same year as SAT on the log-scale.
	(SNAOC) Estimate of the sum of sulphate, nitrate, ammonium and organic carbon simulated using the GEOS Chem chemical transport model.
	(DST) Estimate of compositional concentrations of mineral dust simulated using the GEOS Chem chemical transport model.
	(EDxDU) The log of the elevation difference between the elevation at the ground measurement location and the mean elevation within the GEOS Chem simulation grid cell multiplied by the inverse distance to the nearest urban land surface.
Discrete explanatory variables	(LOC) Binary variable indicating whether exact location of ground measurement is known.
	(TYPE) Binary variable indicating whether exact type of ground monitor is known.
	(CONV) Binary variable indicating whether ground measurement is $PM_{2.5}$ or converted from PM_{10}.
Random Effects	Regional temporal (random walk) hierarchical random-effects on the intercept.
	Regional hierarchical random-effects for the coefficient associated with SAT.
	Regional hierarchical random-effects for the coefficient associated with POP.
	Smoothed, spatially varying random-effects for the intercept.
	Smoothed, spatially varying random-effects for the coefficient associated with SAT.
Interactions	Interactions between the binary variables and the effects of SAT.

In GBD 2019, one set of cause-specific risk curves were created for both household air pollution and ambient air pollution as two different sources of $PM_{2.5}$. The burden attributable to $PM_{2.5}$ was estimated for Ischemic Heart Disease, stroke (ischemic and hemorrhagic), COPD, lung cancer, acute lower respiratory infection, and Type II Diabetes, with addition of adverse birth outcomes including low birthweight and short gestation in GBD 2019. A mediation analysis was performed, in which a proportion of the burden attributable to low birthweight and short gestation was attributed to $PM_{2.5}$ pollution since these are already risk factors (and not outcomes) in the GBD. For the six non-mediated outcomes, results from cohort and case-control studies of ambient $PM_{2.5}$ pollution, cohort studies, case-control studies, and randomised-controlled trials of household use of solid fuel for cooking, and cohort and case-control studies of secondhand smoke were used.

For GBD 2019, several important changes to the risk functions were made. Previously, relative risk estimates for active smoking were used, converting cigarettes-per-day to $PM_{2.5}$ exposure in order to estimate the $PM_{2.5}$ relative risk at the highest end of the $PM_{2.5}$ exposure-response curve. For the first time in GBD 2019, active smoking data in the risk curves is not used because with the recent publication of studies in China, India and other higher-exposure settings and additional studies of HAP, it is now possible to include more estimates at high $PM_{2.5}$ levels in the model.[7,8,9,10,11] Furthermore, in contrast to previous cycles of the GBD where the power function used to develop the IER required the inclusion of active smoking data to anchor the risk function, with the current use of splines and their flexibility, it is easier to fit functions to the (ambient, household, and SHS) data without active smoking data. Removal of active smoking information removes an important source of uncertainty in the earlier estimates related to differences in dose rates and other aspects of exposure between active smoking and the other $PM_{2.5}$ sources, including differences in voluntary (active smoking) and involuntary (ambient and household $PM_{2.5}$, secondhand smoke) exposure.[12,13]

Additionally, in the past, the curves for ischemic heart disease and stroke were built based on studies of mortality and used evidence from three studies of both mortality and incidence to scale down the mortality curves to generate estimates of incidence risk. In GBD 2019 incidence and mortality were extracted from all available studies and was included as a covariate in the model. There was no significant difference between estimates of incidence risk and mortality risk, so both types of risk estimates were included in the curve fitting and the same curve was used for both incidence and mortality. This was done for all other outcomes in GBD 2019 as in the past.

For cardiovascular diseases, evidence suggests that the relative risk decreases with age.[14] To account for this in the model, unique risk curves were generated for every five-year age group from 25–29 years to 95

years and older for both ischemic heart disease and stroke. Because the risk data for every unique age group is not available, each study was adjusted based on the median age during follow-up to generate a full adjusted dataset for every curve. The median age of follow-up was calculated by taking the median (or mean) age at enrollment and adding one-half of median or mean follow-up time. If follow-up time was not available, 70% of total study period was taken based on the observed ratio of follow-up time to total study period for other studies. Using the median age during follow-up, each study was extrapolated to the full set of ages where the estimated data point for age was calculated.

In GBD 2019, MRBRT splines were used to fit the risk data with a more flexible shape. While previously TMREL estimates were built into the model fitting, in GBD 2019 the curves were fitted beginning at zero exposure and the TMREL was incorporated into the relative risk calculation process. This allows others to use these risk curves with different counterfactual level of interest to them. The TMREL was assigned a uniform distribution with lower/upper bounds given by the average of the minimum and fifth percentiles of outdoor air pollution cohort studies exposure distributions conducted in North America, with the assumption that current evidence was insufficient to precisely characterize the shape of the concentration-response function below the fifth percentile of the exposure distributions. The TMREL was defined as a uniform distribution rather than a fixed value in order to represent the uncertainty regarding the level at which the scientific evidence was consistent with adverse effects of exposure. The specific outdoor air pollution cohort studies selected for this averaging were based on the criteria that their fifth percentiles were less than that of the American Cancer Society Cancer Prevention II (CPSII) cohort's fifth percentile of 8.2 based on Turner and colleagues (2016).[15] This criterion was selected since GBD 2010 used the minimum, 5.8, and fifth percentile solely from the CPS II cohort. The resulting lower/upper bounds of the distribution for GBD 2019 were 2.4 and 5.9.

When fitting the risk curves, the published relative risk over a range of exposure data were considered. For OAP studies, the relative risk informs the curve from the fifth to the 95th percentile of observed exposure. When this is not available in the published study, the distribution was estimated from the provided information (mean and standard deviation, mean and IQR, etc.). The RR was scaled to this range. For HAP studies, each study was allowed to inform the curve from the ExpOAP to ExpOAP+ExpHAP, where ExpOAP is the GBD 2017 estimate of the ambient exposure level in the study location and year, and ExpHAP is the GBD 2017 estimate of the excess exposure for those who use solid fuel for cooking in the study location and year. For SHS studies, the strategy of exposure estimation was updated in GBD 2019 to also account for outdoor exposure. Similar to the approach used for HAP, each study was allowed to inform the curve from the ExpOAP to ExpOAP+ExpSHS, where ExpOAP is the GBD 2017 estimate of the ambient exposure level in the study location and year, and ExpSHS is an estimate of the excess exposure for those who experience secondhand smoke. This is estimated from the number of cigarettes smoked per smoker per day in a given location and year estimated from a study in Sweden, which measured the $PM_{2.5}$ exposure in homes of smokers.[16] The household $PM_{2.5}$ exposure level was divided by the average number of cigarettes smoked per smoker per day in Sweden over the study duration to estimate the SHS $PM_{2.5}$ exposure per cigarette (2.31 μg/m3 [95% UI 1.53–3.39]). To calculate ExpSHS the estimated number of cigarettes per smoker per day was multiplied by the average $PM_{2.5}$ exposures per cigarette to generate a predicted $PM_{2.5}$ exposure level.

MR-BRT risk splines

Splines on the datasets were fit including studies of OAP, HAP, and SHS using the following functional form, where X and X_{CF} represent the range of exposure characterised by the effect size:

$$MRBRT(X) - MRBRT(X_{CF}) \sim Shift$$

For each of the risk-outcome pairs, various model settings and priors were tested in fitting the MR-BRT splines. The final models used third-order splines with two interior knots and a constraint on the right- most segment, forcing the fit to be linear rather than cubic. An ensemble approach was used to knot placement, wherein 100 different models were run with randomly placed knots and then combined by weighting based on a measure of fit that penalises excessive changes in the third derivative of the curve. Knots were free to be placed anywhere within the fifth and 95th percentile of the data, as long as a minimum width of 10% of

that domain exists between them. Shape constraints were included so that the risk curves were concave down and monotonically increasing, the most biologically plausible shape for the $PM_{2.5}$ risk curve. On the non-linear segments, a Gaussian prior on the third derivative of mean 0 and variance 0.01 was included to prevent over-fitting; on the linear segment, a stronger prior of mean 0 and variance 1e-6 was used to ensure that the risk curves do not continue to increase beyond the range of the data.

For chronic obstructive pulmonary disease, a looser Gaussian prior of mean 0 and variance 1e-4 was used on the linear segment of the risk function. For this outcome, epidemiological evidence was available from household air pollution that the risk continues to increase at higher levels of $PM_{2.5}$.

Low birthweight and short gestation mediation analysis

The outcomes of low birthweight and short gestation include mortality due to diarrhoeal diseases, lower respiratory infections, upper respiratory infections, otitis media, meningitis, encephalitis, neonatal preterm birth, neonatal encephalopathy due to birth asphyxia and trauma, neonatal sepsis and other neonatal infections, haemolytic disease and other neonatal jaundice, and other neonatal disorders. The attributable YLDs for neonatal preterm birth were also calculated. These are specific to ages 0-6 days and 7-27 days. A systematic review of all cohort, case-control, or randomised-controlled trial studies of ambient $PM_{2.5}$ pollution or household air pollution and birthweight or gestational age outcomes. Outcomes measured included continuous birthweight (bw), continuous gestational age (ga), low birthweight (LBW) (<2500 g), preterm birth (PTB) (<37 weeks), and very preterm birth (VPTB) (<32 weeks). Any papers published until March 31, 2018 were included.

Because birthweight and gestational age were modelled using a continuous joint distribution for the GBD, we were interested in how those distributions changed under the influence of $PM_{2.5}$ pollution. Therefore, the continuous shift in birthweight (bw, in grams) and gestational age (ga, in weeks) were estimated at a given $PM_{2.5}$ exposure level. When available, estimates of continuous shift in bw or ga were used directly from each study. When that was not available, the published OR/RR/HR for LBW, PTB, or VPTB were used and the following strategy:

1. Extract the OR/RR/HR from the study.
2. Select the GBD 2017 estimated bw-ga joint distribution for the study location and year.
3. Calculate the number of grams or weeks required to shift the distribution such that the proportion of births under the specified threshold (P) is reduced by the study effect size to a counterfactual level (Pcf).
4. Save the resulting shift and 95% CI as the continuous effect.

A MR-BRT spline was fit to these studies, where the difference in the value of the model at the upper concentration (X) and the value of the model at the counterfactual concentration (X_{CF}) was equal to the published or calculated shift in bw or ga. The same model and priors as the non-mediated outcomes were fit, except for COPD, because the change in birthweight and gestational age was expected to be negative, the shape constraints were monotonically decreasing and concave up.

$$MRBRT(X) - MRBRT(X_{CF}) \sim Shift$$

Once the curves of estimated shifts were obtained across the exposure range, the shift in both birthweight and gestational age for total female particulate matter pollution exposure were estimated in each location and year. Because the epidemiological studies mutually controlled for birthweight and gestational age, these shifts were assumed to be independent, the observed distributions were then shifted to reflect the expected bwga distribution in the absence of particulate matter pollution.
These shifted distributions were used as the counterfactual in the population attributable fractions (PAF) calculation equation to calculate the burden attributable to $PM_{2.5}$ pollution. To calculate PAFs, the distribution was divided into 56 bw-ga categories, each with a unique RR. Let p_i be the observed proportion of babies in category, i and p_i' be the counterfactual proportion of babies in category, i if there were no particulate matter pollution.

$$PAF_{PM} = \frac{\sum_{i\,\in bwga\ category} RR_i p_i - \sum_{i\in bwga\ category} RR_i p_i'}{\sum_{i\,\in bwga} RR_i p_i}$$

This PAF was proportionately split to ambient and HAP based on exposure as described below. The shift in bw and ga was assumed to be linear across the bwga distribution.

For lower respiratory infections, PAFs attributable to $PM_{2.5}$ were directly estimated in addition to those mediated through birthweight and gestational age. It is expected that some of the directly estimated PAFs are mediated through bw and ga. Additionally, the directly estimated PAF is based on a summary of relative risks for all children under 5 years, so there is a chance that the mediated PAF, which is more finely resolved, could be greater. To avoid double-counting for these two age groups (0-6 days and 0-27 days), the max of the two PAF estimates were considered. If the directly estimated PAF was greater than the bw-ga-mediated PAF, the direct estimate were taken, and if the mediated PAF is greater, the mediated estimates were taken.

PTB incidence and mortality are both outcomes measured in the GBD. 100% of the burden for this cause is attributable to short gestation. To calculate the percentage attributable to particulate matter pollution, the percentage of babies born at less than 37 weeks (*ptb*) and the percentage of babies that would have been born at less than 37 weeks in the counterfactual scenario of no particulate matter pollution (*ptb'*) were estimated.

$$PAF_{ptb,pm} = 1 - \frac{p_{ptb}'}{p_{ptb}}$$

B. Household air pollution

Exposure to household air pollution from solid fuels (HAP) is estimated from both the proportion of individuals using solid cooking fuels and the level of $PM_{2.5}$ air pollution exposure for these individuals. Solid fuels in this analysis include coal, wood, charcoal, dung, and agricultural residues.

Data

Data sources on HAP from solid fuel use in India include national health surveys such as the National Family Health Survey and the District Level Household Survey, nationwide surveys of the National Sample Survey Organisation, and the Census of India, as well as other published and unpublished epidemiological studies.

Globally, information on use of solid fuels were extracted from the standard multi-country survey series such as Demographic and Health Surveys (DHS), Living Standards Measurement Surveys (LSMS), Multiple Indicator Cluster Surveys (MICS), and World Health Surveys (WHS), as well as censuses and country-specific survey series. To fill the gaps of data in surveys and censuses, updated estimates from WHO Energy Database and those extracted from literature through systematic review were used. Each nationally or sub nationally representative data point provided an estimate for the percentage of households using solid cooking fuels. The studies from 1980 to 2019 were used to inform the time series. The sources that did not distinguish specific primary fuel types, estimated fuel used for purposes other than cooking (eg, lighting or heating), failed to report standard error or sample size; had over 15% of households with missing responses, reported fuel use in physical units, or were secondary sources referencing primary analyses were excluded from the analyses.

Many estimates in the WHO Energy Database and other reports quantify the proportion of households using solid fuel for cooking; however, for this analyses the proportion of individuals using solid fuel for cooking was to be used. To crosswalk these estimates, the fuel use at both the individual and household levels were extracted. 3676 source-specific pairs were included in the MRBRT crosswalk model.

MR-BRT crosswalk adjustment factors for household air pollution exposure

Data input	Reference or alternative case definition	Gamma	Beta coefficient, logit (95% CI)
Proportion of individuals	Ref	0.097	--
Proportion of households	Alt		-0.095 (-0.100, -0.090)

This coefficient was then applied to household-only reports with the following formula:

$prop_{individ}$ = the proportion of individuals using solid fuel for cooking, and

$prop_{hh}$ = the proportion of households using solid fuel for cooking.

$$\log\left(\frac{prop_{individ}}{1 - prop_{individ}}\right) = \log\left(\frac{prop_{hh}}{1 - prop_{hh}}\right) - \beta$$

or

$$prop_{individ} = \frac{prop_{hh} * e^{-\beta}}{1 - prop_{hh} + prop_{hh} * e^{-\beta}}$$

The effect was that the household studies were inflated to account for bias. Larger households were more likely to use solid fuel for cooking. The crosswalk model was informed by 3,676 data points and 10% of the studies were trimmed as outliers.

Modelling strategy

Household air pollution was modelled at household level using a three-step modelling strategy (ST-GPR) that uses linear regression, spatiotemporal regression, and Gaussian process regression (GPR).

The first step was a mixed-effect linear regression of logit-transformed proportion of households using solid cooking fuels. The linear model contains maternal education and the proportion of population living in urban areas as covariates and has nested random effects by GBD region and GBD super-region. The full ST-GPR process is specified elsewhere.[2,17] No substantial modelling changes were made in this round compared to GBD 2017.

In addition to the previously included outcomes of lower respiratory infections (LRI), stroke, ischemic heart disease (IHD), chronic obstructive pulmonary disease (COPD), lung cancer, type 2 diabetes, and cataract, in GBD 2019 low birthweight and short gestation was added as a new outcome of household air pollution through a mediation analyses.

Prior to GBD 2019, the results of an external meta-analysis for cataracts were utilised with a summary relative of 2.47 with 95% CI (1.63 - 3.73).[18] While this effect estimate was for both sexes, in the past burden was estimated for women only because women are known to have higher HAP exposure than men. In GBD 2019, a meta-regression analysis of household air pollution and cataracts was performed. All of the component studies of the above meta-analysis paper were extracted and included, except one cross-sectional study. GBD risk factor analyses typically do not include cross-sectional analyses. In an updated literature search, one additional paper describing different fuel types and cataracts was found.[5] This study was also excluded because there was no comparison group without solid fuel use. The resulting dataset contained eight estimates from six sources in India and Nepal.

On these eight estimates, a MR BRT meta-regression was run to generate a summary effect size of 2.51 (1.58 - 3.96). A study-level bias covariate of whether or not the study participants were blind to the exposure-outcome pair of interest was included. The prior on this covariate was a Gaussian distribution with mean 0 and variance 0.1. The prior on gamma was a Gaussian distribution with mean 0.04 and 0.1. The table and figure below provide the model coefficients and a visual representation.

MR-BRT relative risk meta-analysis for household air pollution and cataract

Covariate	Gamma	Beta coefficient, logit (95% CI)	Beta coefficient, adjusted (95% CI)
Intercept	0.40	0.918 (0.460, 1.377)	2.51 (1.58, 3.96)
Outcome unblinded		0.031 (-0.450, 0.512)	1.03 (0.64, 1.67)

Studies reported effect sizes for males, females, and/or both sexes. In a sensitivity analysis a covariate for sex was included and it was found that there was no significant difference in effect size by sex. Therefore, cataract is now estimated as an outcome of household air pollution in both males and females.

In GBD 2019, substantial changes were also made to particulate matter risk curves. These risk curves, utilising splines in MR-BRT, the new mediation analysis with birthweight and gestational age, and the joint-estimation PAF approach as described in the ambient particulate matter section of this appendix. The TMREL is defined as uniform distribution between 2.4 and 5.9 ug/m3 $PM_{2.5}$.

In order to use the particulate matter risk curves, the level of exposure to particulate matter with diameter of less than 2.5 micrometers ($PM_{2.5}$) were estimated for individuals using solid fuels for cooking. The Global Household Air Pollution (HAP) Measurements database from WHO contains 196 studies with measurements from 43 countries of various pollution metrics in households using solid fuel for cooking.[18] From this database, all measurements of $PM_{2.5}$ using indoor or personal monitors were taken. In addition to the WHO database, eight additional studies from a systematic review conducted in 2015 for GBD were also included. The final dataset included 336 estimates from 75 studies in 43 unique locations. 260, 64, nine, and three measurements indoors, on personal monitors for females, children (under 5), and males were included, respectively. 274 estimates were in households using solid fuels, 47 in households only using clean (gas or electricity) fuels, and 15 in households using a mixture of solid and clean fuels. The following model was used:

$$\log(excess\ PM) \sim solid + measure\ group + 24\ hr\ measurement + SDI + (1|study)$$

Where,

- 24-hour measurement: binary variable equal to 1 if the measurement occurred over at least a 24-hour period and not only during mealtimes
- Measure group: categorical variable indicating indoor, female, male, or children
- Solid: indicator variable equal to 1 if the measurements were among households using solid fuel only, 0.5 if the measurements represented a mix of clean and solid fuels, and 0 if the households only used clean fuels.

The Socio-demographic Index (SDI) was also included as a variable to predict a unique value of HAP for each location and year based on development along with a random effect on study. SDI is a composite indicator of development status, which ranges from 0 to 1, and is a geometric mean of the values of the indices of lag-distributed per capita income, mean education in people aged 15 years or older, and total fertility rate in people younger than 25 years in the state. Each study was weighted by its sample size. Before modelling, the excess particulate matter in households using solid fuel was calculated by subtracting off the predicted ambient $PM_{2.5}$ value in the study location and year based on the GBD 2017 $PM_{2.5}$ exposure model. The final model coefficients are included below:

Variable	Beta, log (95% CI)	Beta, adjusted (95% CI)
Intercept	6.23 (4.58, 7.88)	506 (97, 2635)
Solid	2.60 (2.06, 3.13)	13.4 (7.8, 23.0)
Measure group		
o Indoor (ref)	-0.56 (-1.15, 0.04)	0.57 (0.32, 1.04)
o Female	-	-
o Male	-1.56 (-3.81, 0.70)	0.21 (0.02, 2.02)
o Child	-1.13 (-2.06, -0.20)	0.32 (0.13, 0.82)
24-hour measurement	-0.29 (-1.04, 0.46)	0.75 (0.35, 1.59)
SDI	-6.42 (-9.30, -3.54)	1.6 e -3 (9.1 e -5, 2.9 e -2)

Therefore, for females in households using solid fuel, the long-term mean excess $PM_{2.5}$ exposures due to the use of solid fuels is expected to be 1,522, 117, and 9 µg/m3 in SDI of 0.1, 0.5, and 0.9, respectively.

Because there are so few studies of personal monitoring in men and children, rather than directly using the results of the model, ratios were generated using studies that measured at least two of the population groups for any size particulate matter. For $PM_{2.5}$ the predicted ambient $PM_{2.5}$ value was estimated in the study location and year based on the GBD 2017 $PM_{2.5}$ exposure model as the "outdoor" measurement, and for PM_4 and PM_{10} published values in the studies themselves were used. This outdoor value was first subtracted off from each PM measurement, and then calculated the ratio of male to female and child to female exposure, weighted by sample size.

Study	Location	Year	Pollutant	Female N	Female PM	Group	N	PM	Outdoor
Balakrishnan et al., 2004	Andhra Pradesh, Rural	2004	PM_4	591	352	male	503	187	94
Gao X et al., 2009.	Tibet	2009	$PM_{2.5}$	52	127	male	85	111	27
Dasgupta et al., 2006	Bangladesh	2006	PM_{10}	944	209	male	944	166	50
Devkumar et al., 2014	Nepal	2014	$PM_{2.5}$	405	169	male	429	167	90
Balakrishnan et al., 2004	Andhra Pradesh, Rural	2004	PM_4	591	352	child	56	262	94
Dionisio et al., 2008.	The Gambia	2008	$PM_{2.5}$	13	275	child	13	219	31
Dasgupta et al., 2006	Bangladesh	2006	PM_{10}	944	209	child	944	199	50

The final ratios were 0.64 95% CI (0.45-0.91) for males and 0.85 95% CI (0.56-1.31) for children. These results were used to scale the $PM_{2.5}$ mapping model for these age and sex groups to input into the $PM_{2.5}$ risk curves.

C. Ambient ozone pollution

Exposure to ozone pollution was defined as the seasonal (6-month period with highest mean) 8 hour daily maximum ozone concentrations, measured in parts per billion (ppb). To estimate the distribution of exposure to ozone in ambient air for the years 1990 to 2017, ozone ground measurement data were combined with chemical transport model estimate using Bayesian maximum entropy.

Data

Ozone monitoring data were taken from the Tropospheric Ozone Assessment Report (TOAR) – Phase 1, which contains data from seven sites in India for surface ozone metrics.[19] Since the TOAR data are available publically only until 2015, an update was made to include readily available TOAR datasets until 2017. All observations were processed to provide the six-month ozone season average of eight-hour daily maximum ozone concentrations.

Modelling strategy

A combination of global atmospheric chemical transport models was used, many of which simulated specified dynamics for the Chemistry-Climate Model Initiative (CCMI).[20] The eight models and years available include CHASER (1990– 2010), MOCAGE (1988–2016), MRI-ESM (1988–2017), NASA MERRA2-GMI (1988–2017), NCAR CESMChem (1988–2010), NCAR WACCM (1988–2010), GFDL AM3 (1988–2014), and GFDL AM4 (2010–2016).

These models provided hourly ozone data, which was used to calculate the six-month maximum daily eight-hour maximum ozone mixing ratio (ppb). A multi-model composite of the specified-dynamics models in each year from 1990 to 2017 was created using the M^3Fusion method.[21] A linear combination of models was produced for each year using this multi-model composite that minimizes the mean square error as compared to the observations in each world region and it corrects to minimize the mean model bias in each region. In this process each model in every region was weighted to minimize the difference between the multi-model average and observations.

Regions with sparse data was taken into account, as the M^3Fusion method relies on surface measurements to change the weights. North America and Europe use weights-based model and observation values for each individual year. The rest of the world regions (South America, Africa, south central Asia, east Asia, Russia, and Oceania) use individual year weights for 2000–2010, and apply weights calculated from the

aggregated 2000 to 2010 period for 1990–1999. For 2011–2017, east Asia used individual year weights, while South America, Africa, south central Asia, Russia, Oceania, and Antarctica use weights from the aggregated 2011–2014 period.

A geo-statistical modelling tool named “Bayesian Maximum Entropy (BME)” was used to combine various knowledge bases for an air pollutant to create a single product. The BME model was used to combine site-specific measurements and model concentrations, making use of the correlations between measurement locations. This modeling uses the measurement values to correct the M^3Fusion Model locally around each station spatially and temporally, allowing future and past observations to provide input. Using this modeling more measurement locations became available through time and thus this method allows later measurements to influence ozone surfaces earlier in the period, which is particularly important in China and data-sparse regions. As part of the BME modelling, the range over which each measurement can correct the M^3Fusion Model and how each measurement’s impact decreases over distance in time and space are calculated. Further than combining these knowledge bases to produce an estimate of ozone pollution, BME modeling estimates a variance, which can be used to assess estimation confidence at different locations.

This results were calculated at 0.5° resolution and the NASA G5NR-Chem model was used to downscale estimates at finer resolution. This model simulates surface ozone concentrations at 0.125° by 0.125° resolution for July 2013 to June 2014.[22] The G5NR-Chem model were re-gridded from 0.125° resolution to 0.1° resolution. Even though the raw values for 2013–2014 is not expected to hold true for every year, it is believed that the spatial distribution of this model can be used to inform the fine-scale spatial pattern for each year. Following steps were performed to add fine resolution.

- Regrid NASA G5NR-Chem from 0.125° resolution to 0.1° resolution.
- Average each 0.5° NASA G5NR-Chem grid cell.
- Calculate the difference between BME estimation and the average NASA G5NR-Chem at 0.5°.
- Add the calculated difference to NASA G5NR-Chem at 0.1° to obtain BME estimation at 0.1°.

Adding fine resolution to the results keeps the average of each 0.5° grid cell the same as the original estimation at 0.5°, as well as the global average.

To estimate global ozone in 2018 and 2019, for each 0.1° grid cell, a log-linear model of the ozone estimates on year was run for the most recent ten years (2008–2017) of the following form:

$$\log(\text{ozone}) \sim \text{year}+1.$$

Splines were considered for predicting the estimates, but due to annual variation of ozone, a log-linear trend was found to provide the most reasonable prediction. Since long-term trends and effects provided the most reasonable prediction than annual variation, a three-year mean of exposure centered on the year of interest was used for burden estimation during the years 1991–2016. This estimation strategy is in line with the estimation methodology for ambient particulate matter air pollution. Since 1989 and 2018 data were not available in the estimates, two year means of (1990/1991) and (2016/2017) were used for the years 1990 and 2017 respectively.

A conservative estimate of the variance was made to estimate the variance for the three-year mean to generate confidence intervals as the information on the covariance between years was not available.

The GBD 2019 method for ozone exposure estimates improves upon the GBD 2017[1] as follows.

- GBD 2017 estimates used observations in a specific year to correct the model within 2° of a monitoring station. However, the radius of influence of each observation was used in GBD 2019, which is defined by the spatial covariance and this covariance shows that much of the influence of an observation is lost after 1°.
- GBD 2019 modeling considered the bias-correction estimates for the year in which they were observed and also influence other year estimates according to the temporal covariance modeling. This is important for regions that were not monitored over the entire 1990–2017 time-period.

- In GBD 2019 estimates, the fine spatial structure of the final product represents the spatial distribution of the 0.125° NASA G5NR-Chem model.

Theoretical minimum-risk exposure level

Theoretical minimum-risk exposure level (TMREL) was based on the exposure distribution from the ACS CPS-II study[15] and is uniform distribution around the minimum and fifth percentile values observed in the cohort, ~U (29.1, 35.7), in ppb.

Relative risks

COPD mortality is the only included outcome for ambient ozone pollution. In GBD 2017, a literature review of studies examining long-term ozone exposure and COPD was performed. Five cohorts from Canada, the UK, and the USA were included,[15,23,24] all of which reported ozone effects on COPD mortality. For this reason, GBD only included mortality and not incidence as an outcome of ozone exposure.

In GBD 2019, methodology was updated to use MR-BRT for the meta-analysis of relative risks. Because GBD had only five data points and no study-level covariates priors were included. The inverse-variance weighted meta-analysis of the five cohorts provided an estimated relative risk of 1.06 (95% CI 1.03, 1.10) with an estimated gamma (including between study heterogeneity) of 0.004.

Population attributable fraction

The PAFs were calculated at the grid-cell level and aggregated up to GBD locations using population data from the Gridded Population of the World database. Different version of model was used for all years except for the years 1990 and 1995.[1]

References

1. GBD 2019 Risk Factors Collaborators. Global burden of 87 risk factors in 204 countries and territories, 1990–2019: a systematic analysis for the Global Burden of Disease Study 2019. *The Lancet* 2020; 396: 1223–49.
2. India State-Level Disease Burden Initiative Air Pollution Collaborators. The impact of air pollution on deaths, disease burden, and life expectancy across the states of India: the Global Burden of Disease Study 2017. *Lancet Planet Health* 2019; 3: e26-e39.
3. van Donkelaar A, Martin RV, Brauer M, Hsu NC, Kahn RA, Levy RC, et al. Global estimates of fine particulate matter using a combined geophysical-statistical method with information from satellites, models, and monitors. *Environ Sci Technol* 2016;50: 3762–72.
4. Rue H, Martino S, Chopin N. Approximate Bayesian inference for latent Gaussian models by using integrated nested Laplace approximations. *J. R. Statist. Soc* 2009;71: 319–92.
5. Thomas ML, Shaddick G, Simpson D, de Hoogh K, Zidek JV. Data integration for high-resolution, continental-scale estimation of air pollution concentrations. 2019. http://arxiv.org/abs/1907.00093
6. Generalized Additive Models: An Introduction with R, Second Edition. CRC Press; 2017. Available from: https://www.routledge.com/Generalized-Additive-Models-An-Introduction-with-R-Second-Edition/Wood/p/book/9781498728331
7. Yin P, Brauer M, Cohen A, Burnett RT, Liu J, Liu Y, et al. Long-term fine particulate matter exposure and nonaccidental and cause-specific mortality in a large national cohort of Chinese men. *Environ Health Perspect*. 2017 07;125: 117002.
8. Li T, Zhang Y, Wang J, et al. All-cause mortality risk associated with long-term exposure to ambient PM2·5 in China: a cohort study. *Lancet Public Health* 2018; 3: e470–7.
9. Yang Y, Tang R, Qiu H, et al. Long term exposure to air pollution and mortality in an elderly cohort in Hong Kong. *Environ Int* 2018; 117: 99–106.
10. Hystad P, Larkin A, Rangarajan S, AlHabib KF, Avezum A, Tumerdem Calik KB, et al. Outdoor fine particulate matter air pollution and cardiovascular disease: results from 747 communities across 21 countries in the PURE Study. *Lancet Glob Health* [in press].

11. Yusuf S, Joseph P, Rangarajan S, Islam S, Mente A, Hystad P, et al. Modifiable risk factors, cardiovascular disease, and mortality in 155 722 individuals from 21 high-income, middle-income, and low-income countries (PURE): a prospective cohort study. *The Lancet* 2020;395: 795–808.
12. Burnett RT, Pope CA, Ezzati M, Olives C, Lim SS, Mehta S, et al. An integrated risk function for estimating the global burden of disease attributable to ambient fine particulate matter exposure. *Environ Health Perspect* 2014;122: 397–403.
13. Pope CA, Cohen AJ, Burnett RT. Cardiovascular disease and fine particulate matter: lessons and limitations of an integrated exposure response approach. *Circ Res* 2018;122: 1645–7.
14. Lind L, Sundström J, Ärnlöv J, Lampa E. Impact of aging on the strength of cardiovascular risk factors: a longitudinal study over 40 years. *J Am Heart Assoc* 2018;7: e007061.
15. Turner MC, Jerrett M, Pope CA, Krewski D, Gapstur SM, Diver WR, et al. Long-Term Ozone Exposure and Mortality in a Large Prospective Study. *Am J Respir Crit Care Med* 2016; 193:1134–42.
16. Semple S, Apsley A, Azmina Ibrahim T, Turner SW, Cherrie JW. Fine particulate matter concentrations in smoking households: just how much secondhand smoke do you breathe in if you live with a smoker who smokes indoors? *Tob Control* 2015;24: e205-211.
17. Smith KR, Bruce N, Balakrishnan K, Adair-Rohani H, Balmes J, Chafe Z, et al. Millions dead: how do we know and what does it mean? methods used in the comparative risk assessment of household air pollution. *Annu Rev Public Health* 2014; 35:185–206.
18. Shupler M, Balakrishnan K, Ghosh S, Thangavel G, Stroud-Drinkwater S, Adair-Rohani H, et al. Global household air pollution database: kitchen concentrations and personal exposures of particulate matter and carbon monoxide. *Data Brief* 2018;21: 1292–5.
19. Schultz MG, Schröder S, Lyapina O, Cooper O, Galbally I, Petropavlovskikh I, et al. Tropospheric Ozone Assessment Report: database and metrics data of global surface ozone observations. *Elem Sci Anth* 2017; 5:58.
20. Morgenstern O, Hegglin MI, Rozanov E, O'Connor FM, Abraham NL, Akiyoshi H, et al. Review of the global models used within phase 1 of the Chemistry–Climate Model Initiative (CCMI). *Geosci Model Dev* 2017;10: 639–71.
21. Chang K-L, Cooper O, West JJ, Serre ML, Schultz M, Lin M, et al. A new method (M 3 Fusion v1) for combining observations and multiple model output for an improved estimate of the global surface ozone distribution. *Geosci Model Dev* 2019;12: 955–78.
22. Hu L, Keller CA, Long MS, Sherwen T, Auer B, Da Silva A, et al. Global simulation of tropospheric chemistry at 12.5 km resolution: performance and evaluation of the GEOS-Chem chemical module (v10-1) within the NASA GEOS Earth system model (GEOS-5 ESM). *Geosci Model Dev* 2018;11: 4603–20.
23. Carey IM, Atkinson RW, Kent AJ, van Staa T, Cook DG, Anderson HR. Mortality associations with long-term exposure to outdoor air pollution in a national English cohort. *Am J Respir Crit Care Med* 2013;187: 1226–33.
24. Cox Proportional Survival Model Hazard Ratios from Census Years (1991,1996,2001) to 2011 for Adults Aged 25 to 89 in CanCHEC Cohort. [Data shared for this analysis].

D. Uncertainty intervals

Point estimates for each quantity of interest were derived from the mean of the draws, while 95% uncertainty intervals (UIs) were derived from the 2.5th and 97.5th percentiles of the 1000 draw level values. Uncertainty in the estimation is attributable to sample size variability within data sources, different availability of data by age, sex, year, or location, and cause specific model specifications. We determined UIs for components of cause-specific estimation based on 1000 draws from the posterior distribution of cause-specific mortality by age, sex, and location for each year included in the GBD 2017 analysis. Similarly, for non-fatal estimates if there was a change in disease estimates between locations or over time that was in the same direction in more than 950 of the 1000 samples we report it as significant. With this approach, uncertainty could be quantified and propagated into the final quantities of interest.

2. Estimation of output losses due to air pollution

Data

To compute Gross State Domestic Product (GSDP) per worker (Y_i/L_i), the per capita GDP (Y_i/N_i) in state I,[1] was divided by the ratio of workers to the population (L_i/N_i).[2] Labor's share of GDP (α) was computed for the country as a whole, based on the Penn World Tables 9.0.[3] The labor's share of GDP at market prices measured in 2014 was multiplied by an adjustment factor that reflects the ratio of GDP at basic prices to GDP at market prices. This adjustment factor was computed from unpublished data obtained from Robert Inklaar on October 13, 2018. This resulted in α = 0.456.

Other parameters that vary by state include the ratio of worker to total population and survival rates. Data from the National Sample Survey 2011-2012 (NSS 68th round)[2] was used to calculate the ratio of worker to total population (L_{ij}/N_{ij}) for each state and age groups between 10 to 84 years. Because only aggregate data are reported for ages 65 and older, (L_{ij}/N_{ij}) for each age over 65 was determined by assuming that the worker-population ratio declines linearly from age 65 to age 85, becoming zero at age 85. The annual survival rate from age j to age t in each state, $\pi_{ij,t}$, was computed from life tables provided by the Global Burden of Disease Study 2019.[4]

The present value of lost output depends on the rate of growth in output per worker *(g)* and the discount rate *(r)*. In the base case, the real rate of growth in output per worker *(g)* was based on historic data from the KLEMS database.[5] The real rate of growth in labor income over the period 1990-2000 to 2016-2017 was 6.47%. Adjusting this for the rate of growth in the labor force over this period[6] yields an annual rate of growth in output per worker of 4.83%. The rate of interest, r, is chosen to be 6%, which is, as of May 2020, the rate of return on 10-year government bond in India. Because it is the ratio of (1+g)/(1+r) that drives the results, all values of g and r that satisfy the equation (1+g)/(1+r) = 0.989 are consistent with our results. For sensitivity analysis we considered the discount rate (r) between 4% and 8%. The output damages from air pollution vary as a function of the discount rate as shown below.

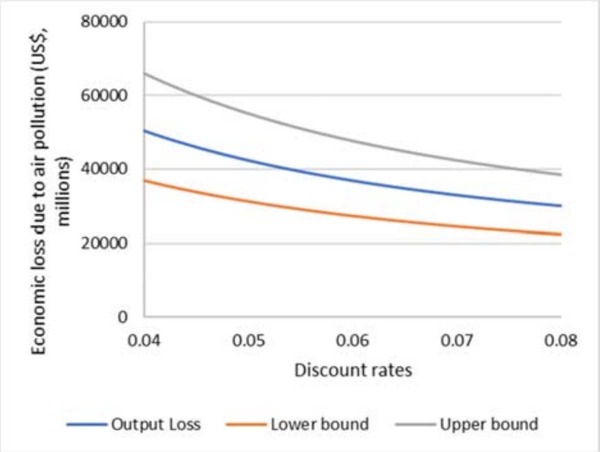

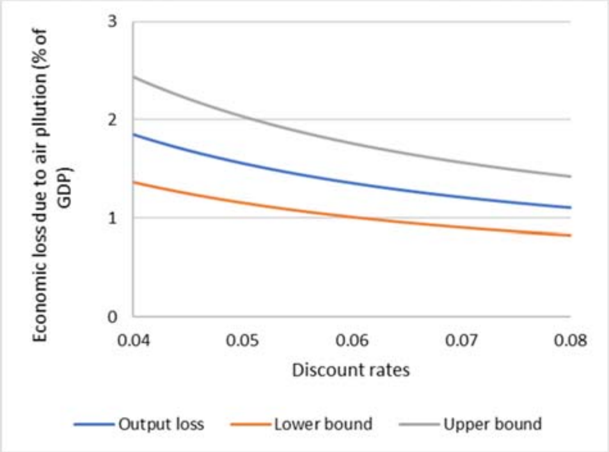

Output losses associated with air pollution mortality

The present discounted value of the loss in Gross Domestic Product (GDP) attributable to mortality associated with $PM_{2.5}$ in 2019 was calculated as follows. The loss in GDP in state i in 2019 if a worker dies is equal to labor's share of GDP (α) multiplied by GDP (Y_i), divided by the number of persons who are employed (L_i). The workers of all ages in a state were assumed to produce the same output per worker. Because not all persons of age j are working, the expected value of GDP per worker for a person of age j (W_{ij2017}) is equal to ($\alpha Y_i/L_i$) times the ratio of the number of workers of age j, L_{ij}, to the population of age j, N_{ij},

$$W_{ij2019} = (\alpha Y_i/L_i)*(L_{ij}/N_{ij}) \qquad (1)$$

In calculating (1) the labor's share of GDP (α) was assumed to be constant across states. Also the ratio of L_{ij}/N_{ij} was assumed to remain constant over time.

To calculate the loss in market and non-market output in 2019 equation (1) was modified to allow for household production. The household production in India is estimated to be 30% of GDP.[7] Therefore, $W'_{ij\,2019}$ was calculated as:

$$W'_{ij2019} = (\alpha Y_i/L_i)^*(L_{ij}/N_{ij}) + \lambda_j(\alpha Y_i/L_i)^*[1-(L_{ij}/N_{ij})] \quad (2)$$

where λ_j represents the fraction of output attributable to non-market production for a person of age j. For children and the older population, $(L_{ij}/N_{ij}) = 0$, so the first term in (2) is zero ($L_{ij}/N_{ij} = 0$ for $j < 10$ and $j > 84$. We also assume that non-market output is zero for children and the older population ($\lambda_j = 0$ for $j < 10$ and $j > 84$. For those aged between 10-84 years λ_j was assumed to be 0.3.

If a person of age j dies in the current year, their contribution to GDP will be lost for all future years of their working life. To compute the value of GDP lost in future years GDP per worker in state i was assumed to grow at rate g. If labor's share of GDP and the fraction of population of working age (L_{ij}/N_{ij}) remain constant for all i and j, this implies that lost GDP at age t of a person currently of age j will equal $(\alpha Y_i/L_i)^*(L_{it}/N_{it})^*(1+g)^{t-j}$. This must be weighted by the probability that an individual would have survived to age t, where $\pi_{ij,t}$ is the probability that a person of age j in state i survives to age t. The loss in GDP in future years is weighted by the probability that an individual who dies this year would have survived to each future year of his working life. The value of GDP lost in the future was discounted at the annual rate r.

Given the previous assumptions, the present discounted value of lost market and non-market output for a person of age j in state i who dies in 2019, PV_{ij}, is:

$$PV_{ij} = \sum_{t=j}^{84} \pi_{ij,t} \left[\left(\frac{L_{it}}{N_{it}}\right)\left(\frac{\alpha Y_i}{L_i}\right) + \lambda_t \left(1 - \frac{L_{it}}{N_{it}}\right)\left(\frac{\alpha Y_i}{L_i}\right)\right] \left(\frac{1+g_i}{1+r_i}\right)^{t-j} \quad (3)$$

Equation (3) was calculated for $j = 0, \ldots, 84$, following the assumptions for λ_t made above.

The total output lost due to air pollution is the product of PV_{ij} and D_{ij}, the number of deaths due to air pollution in 2019 of persons of age j in state i, summed over all j. D_{ij} is computed separately for all air pollution deaths—deaths associated with ambient particulate matter pollution and household air pollution and separately for deaths associated with ambient particulate matter pollution and household air pollution. The confidence intervals for total output lost due to air pollution was calculated using the confidence intervals of estimated deaths attributable to air pollution in GBD 2019.[4]

Output losses associated with air pollution morbidity

The lost output due to morbidity associated with air pollution in 2019 was computed by multiplying the number of years of healthy life lost due to disability (YLDs) associated with air pollution in 2019 by the expected loss in output per person. Results are reported by state and category of air pollution. The expected loss in output per person is given by equation (2) above. The data on YLD associated with air pollution in state i and age j (YLD_{ij}) are taken from the published paper.[4] The output loss associated with morbidity in 2019 for persons of age j in state i, M_{ij} is given by:

$$M_{ij} = W'_{ij2019} * YLD_{ij}$$

Morbidity losses, summed across all age groups, are reported by state and category of pollution (all air pollution, ambient PM pollution and household air pollution). The confidence intervals reported reflect the confidence intervals in YLDs due to air pollution as computed in GBD 2019.[4]

These estimates depend on a number of assumptions, which, if changed, would alter the results. This study assumed that the rate of growth in output per worker (g_i) and the discount rate (r_i) were same for all states. For simplicity, it was assumed that labor's share of GDP remains constant over time at its current value. It was also assumed that the state-specific life tables remain constant over the period of the analysis, which for children is over 80 years. This will understate losses in states with low social development indexes, where survival probabilities are likely to increase in the future.

Finally, the output losses in monetary terms and as a percent of GDP for all deaths and morbidity attributable to ambient particulate matter pollution and household pollution was calculated for every state of India in 2019 using the state-wise India GDP in 2018-19,[1] and for India by aggregating the state estimates.

References

1. Ministry of Statistics and Program Implementation. State Domestic Product and other aggregates, 2011-2012 series. Ministry of Statistics and Program Implementation, Government of India. 2020. http://mospi.nic.in/data (accessed June 12, 2020).
2. National Sample Survey Office, Ministry of Statistics & Programme Implementation, Government of India. Employment and unemployment situation in India: NSS 68 round, July 2011- June 2012. 2020. http://mospi.nic.in/sites/default/files/publication_reports/nss_report_554_31jan14.pdf (accessed June 12, 2020).
3. Feenstra RC, Inklaar R, Timmer MP. The next generation of the Penn world table. *American Economic Review* 2015;105: 3150–82.
4. GBD 2019 Risk Factors Collaborators. Global burden of 87 risk factors in 204 countries and territories, 1990–2019: a systematic analysis for the Global Burden of Disease Study 2019. *The Lancet* 2020; 396: 1223–49.
5. Das DK, Erumban AA, Aggarwal S, Das PC. Measuring productivity at the industry level: the India KLEMS database. 2019;86. https://rbidocs.rbi.org.in/rdocs/PublicationReport/Pdfs/KLEMS6C89D200F2B4464A873C469267506ADA.PDF
6. The World Bank. Labor force, total - India. The World Bank. 2020. https://data.worldbank.org/indicator/SL.TLF.TOTL.IN?locations=IN (accessed June 12, 2020).
7. RN Pandey. Women's contribution to the economy through their unpaid household work. National Institute for Public Finance and Policy Working Paper. 2001 https://www.nipfp.org.in/media/pdf/working_papers/WP_2001_182.pdf (accessed June 12, 2020).

3. $PM_{2.5}$ concentration, use of solid fuels, and ozone concentration in the states of India, 2019

States of India*	Population-weighted mean ambient $PM_{2.5}$ µg/m³ (95% UI)	Percentage of population using solid fuels (95% UI)	Population-weighted incremental $PM_{2.5}$ µg/m³ from use of solid fuels (95% UI)	Population-weighted ozone concentration in parts per billion (95% UI)
India	91.7 (69.6-113.9)	56.3 (55.1-57.4)	82.8 (41.9-153.8)	66.2 (66.0-66.3)
Bihar	167.8 (86.7-258.6)	83.2 (79.6-86.2)	145.9 (72.1-275.9)	70.9 (70.4-71.4)
Uttar Pradesh	182.9 (104.3-284.4)	67.1 (63.1-70.8)	99.8 (51.8-181.8)	69.8 (69.4-70.1)
Manipur	38.4 (28.8-50.2)	56.7 (48.7-63.5)	59.4 (30.3-109.7)	51.3 (49.7-53.1)
Jharkhand	85.8 (55.2-134.1)	79.7 (77.0-82.1)	115.0 (57.5-216.2)	70.1 (69.4-70.8)
Madhya Pradesh	72.8 (45.1-117.6)	69.2 (66.2-71.8)	119.3 (59.0-226.5)	65.4 (65.1-65.7)
Assam	37.0 (25.9-50.9)	72.5 (67.9-76.4)	82.5 (42.8-149.8)	55.7 (55.1-56.3)
Meghalaya	34.0 (26.0-45.0)	72.9 (67.4-77.2)	77.1 (39.7-140.6)	60.7 (59.2-62.3)
Jammu & Kashmir and Ladakh	53.3 (32.0-79.5)	38.8 (30.2-47.4)	55.7 (28.1-103.8)	76.6 (75.8-77.4)
Chhattisgarh	49.8 (31.1-72.4)	75.7 (72.2-78.4)	96.1 (49.5-175.9)	69.1 (68.6-69.7)
West Bengal	77.0 (58.0-99.8)	67.2 (63.3-70.8)	80.4 (41.5-147.5)	68.3 (67.5-69.1)
Nagaland	37.9 (29.8-47.2)	65.3 (60.9-69.4)	50.2 (24.7-94.7)	49.1 (47.1-51.1)
Odisha	46.9 (30.3-66.6)	76.5 (73.7-78.9)	86.1 (44.9-156.8)	71.8 (71.3-72.3)
Rajasthan	89.4 (52.5-129.5)	67.0 (63.4-69.9)	108.6 (55.1-201.5)	58.7 (58.3-59.0)
Tripura	42.7 (32.1-54.6)	63.0 (57.5-68.5)	75.1 (38.8-136.7)	62.4 (60.8-64.0)
Arunachal Pradesh	24.2 (16.1-35.1)	55.1 (49.5-60.4)	78.8 (40.8-143.7)	47.4 (46.3-48.5)
Mizoram	30.6 (23.7-40.3)	34.1 (29.1-39.2)	52.8 (26.3-99.3)	56.2 (53.2-59.3)
Andhra Pradesh	35.7 (27.5-45.1)	37.1 (31.2-43.2)	89.5 (46.3-162.7)	68.4 (67.8-69.0)
Punjab	81.3 (52.4-124.5)	32.5 (27.1-37.7)	50.3 (24.8-94.8)	69.9 (69.3-70.5)
Tamil Nadu	36.9 (23.2-57.1)	27.3 (22.2-32.7)	51.7 (25.6-97.2)	57.6 (56.9-58.3)
Maharashtra	55.3 (43.0-69.1)	35.2 (29.7-40.8)	51.2 (25.3-96.7)	67.4 (66.4-68.4)
Telangana	45.6 (33.0-65.6)	28.0 (25.0-31.2)	79.3 (40.8-145.7)	67.7 (66.4-69.0)
Kerala	15.8 (13.0-18.7)	40.4 (33.0-48.0)	38.0 (17.4-75.0)	56.3 (55.1-57.5)
Himachal Pradesh	36.1 (21.9-52.4)	54.3 (44.9-63.3)	47.2 (23.0-89.6)	72.6 (71.8-73.5)
Karnataka	30.2 (18.1-46.6)	45.6 (39.9-51.2)	68.5 (35.1-125.4)	64.0 (62.9-65.0)
Uttarakhand	65.3 (36.9-104.4)	45.2 (40.3-49.6)	50.9 (25.2-95.8)	67.5 (66.5-68.4)
Gujarat	46.5 (25.3-78.3)	40.9 (35.4-46.2)	57.8 (29.1-107.7)	61.2 (60.2-62.3)
Haryana	123.5 (72.1-184.6)	45.5 (40.3-50.3)	55.8 (28.1-104.4)	65.0 (64.0-66.0)
Other small union territories	47.0 (20.4-97.2)	17.7 (13.9-22.5)	34.5 (15.4-68.9)	65.2 (62.3-67.8)
Sikkim	47.3 (34.7-63.2)	36.6 (30.7-42.5)	49.3 (24.2-93.3)	59.5 (56.7-62.5)
Delhi	217.6 (117.9-297.3)	2.6 (1.6-4.1)	27.6 (11.2-57.5)	63.6 (59.9-67.6)
Goa	22.5 (16.8-39.2)	12.7 (9.9-16.0)	26.2 (10.4-55.4)	63.1 (60.1-66.0)

*The states are listed in the increasing order of per capita GDP in 2018-19.

4. Deaths attributable to air pollution in the states of India, 2019

States of India*	Death rate attributable to air pollution	Number of deaths attributable to air pollution†	Percentage of total deaths attributable to air pollution	Death rate attributable to ambient particulate matter pollution	Number of deaths attributable to ambient particulate matter pollution†	Percentage of total deaths attributable to ambient particulate matter pollution	Death rate attributable to household air pollution	Number of deaths attributable to household air pollution†	Percentage of total deaths attributable to household air pollution	Death rate attributable to ambient ozone pollution	Number of deaths attributable to ambient ozone pollution†	Percentage of total deaths attributable to ambient ozone pollution
**India**	**120 (102-138)**	**1,667,331 (1,415,122-1,924,095)**	**17.8 (15.8-19.5)**	**70 (55-86)**	**979,682 (770,095-1,191,878)**	**10.4 (8.4-12.3)**	**44 (28-62)**	**606,890 (390,625-856,741)**	**6.5 (4.3-9.0)**	12 (6-19)	167,987 (82,017-261,727)	1.8 (0.9-2.7)
Bihar	104 (86-121)	126,460 (105,308-148,310)	18.8 (16.4-21.1)	52 (36-69)	63,273 (43,569-83,704)	9.4 (6.7-12.2)	48 (31-69)	58,816 (37,939-83,622)	8.8 (5.7-12.2)	10 (5-15)	11,920 (5,608-18,571)	1.8 (0.9-2.8)
Uttar Pradesh	144 (118-170)	349,926 (286,430-411,973)	19.5 (16.7-21.8)	90 (68-113)	217,459 (166,319-273,457)	12.1 (9.5-14.5)	47 (28-70)	114,694 (69,184-17,1063)	6.4 (3.9-9.4)	19 (8-30)	45,332 (19,746-73,139)	2.5 (1.1-4.0)
Manipur	78 (62-96)	2,758 (2,187-3,388)	13.9 (12.0-15.8)	46 (31-62)	1,606 (1,083-2,182)	8.1 (5.7-10.5)	31 (18-47)	1,073 (636-1,645)	5.4 (3.3-7.9)	4 (2-6)	132 (58-223)	0.7 (0.3-1.1)
Jharkhand	87 (73-102)	33,136 (27,642-38,553)	16.7 (14.5-18.9)	42 (29-55)	15,880 (11,100-20,734)	8.0 (5.8-10.4)	42 (27-58)	15,923 (10,412-22,084)	8.0 (5.3-11.1)	9 (4-14)	3,345 (1,620-5,190)	1.7 (0.8-2.6)
Madhya Pradesh	126 (104-148)	112,009 (92,397-131,581)	18.7 (16.2-20.8)	60 (44-77)	53,201 (38,893-67,811)	8.9 (6.8-11.0)	61 (40-83)	54,101 (35,439-73,272)	9.0 (6.0-12.2)	12 (6-19)	10,832 (5,072-16,914)	1.8 (0.8-2.8)
Assam	102 (85-121)	36,618 (30,649-43,593)	15.1 (13.2-16.8)	40 (28-53)	14,566 (9,984-18,960)	6.0 (4.2-7.5)	57 (39-78)	20,677 (14,098-28,057)	8.5 (5.9-11.3)	7 (3-11)	2,494 (1,122-4,018)	1.0 (0.5-1.6)
Meghalaya	55 (44-68)	1,874 (1,504-2,321)	11.7 (10.0-14.2)	22 (15-30)	761 (518-1023)	4.8 (3.3-6.2)	30 (21-43)	1,039 (701-1,462)	6.5 (4.4-8.9)	4 (2-7)	132 (60-236)	0.8 (0.4-1.5)
Jammu & Kashmir and Ladakh	114 (94-138)	15,997 (13,108-19,295)	20.2 (17.3-22.7)	73 (56-91)	10,288 (7,818-12,805)	13.0 (10.3-15.5)	29 (16-47)	4,069 (2,256-6,565)	5.1 (2.9-8.1)	20 (9-31)	2,782 (1,296-4,347)	3.5 (1.7-5.2)
Chhattisgarh	131 (110-152)	41,519 (34,890-48,091)	16.9 (14.9-18.8)	55 (38-74)	17,562 (11,957-23,522)	7.2 (5.1-9.4)	71 (48-96)	22,369 (15,316-30,401)	9.1 (6.3-12.2)	10 (5-16)	3,309 (1,545-5,176)	1.3 (0.6-2.1)
West Bengal	123 (101-144)	122,833 (100,633-143,817)	20.8 (18.3-22.8)	71 (52-90)	70,391 (51,677-89,360)	11.9 (9.1-14.4)	48 (30-69)	47,749 (30,228-68,813)	8.1 (5.3-11.3)	10 (5-15)	9,957 (4,890-15,108)	1.7 (0.8-2.6)
Nagaland	65 (52-80)	1,281 (1,016-1,566)	12.6 (11.0-14.2)	34 (23-45)	664 (454-884)	6.5 (4.7-8.2)	30 (18-45)	589 (360-875)	5.8 (3.7-8.2)	2 (1-4)	49 (20-87)	0.5 (0.2-0.8)
Odisha	93 (73-120)	43,409 (33,936-55,732)	12.7 (10.9-15.6)	41 (28-56)	19,113 (13,220-26,014)	5.6 (3.9-7.3)	49 (33-72)	22,854 (1,5162-33,394)	6.7 (4.6-9.1)	6 (3-14)	2,779 (1,184-6,481)	0.8 (0.4-1.9)
Rajasthan	141 (111-169)	113,361 (89,003-135,976)	21.2 (17.2-24.0)	72 (53-93)	58,167 (42,683-74,370)	10.9 (8.3-13.5)	61 (38-88)	49,352 (30,836-71,052)	9.2 (5.9-13.1)	17 (6-27)	2,096 (1,018-3,533)	2.5 (0.9-4.0)
Tripura	122 (98-150)	4,925 (3,944-6,028)	19.0 (16.5-21.3)	62 (43-84)	2,506 (1,738-3,396)	9.7 (7.1-12.5)	54 (34-78)	2,173 (1,381-3,161)	8.4 (5.5-12.0)	11 (5-17)	3,612 (1,638-5,852)	1.8 (0.8-2.6)
Arunachal Pradesh	46 (37-58)	789 (631-999)	11.0 (9.4-13.0)	16 (11-23)	282 (182-395)	4.0 (2.7-5.3)	28 (19-39)	478 (319-673)	6.7 (4.6-9.0)	3 (1-4)	46 (20-76)	0.6 (0.3-1.1)
Mizoram	60 (46-74)	770 (584-949)	11.3 (9.4-13.3)	35 (26-47)	452 (330-602)	6.6 (5.2-8.2)	19 (11-30)	244 (137-378)	3.6 (2.1-5.4)	9 (3-14)	110 (44-177)	1.6 (0.7-2.5)
Andhra Pradesh	116 (93-143)	62,808 (50,176-77,486)	15.6 (13.6-17.6)	58 (40-77)	31,363 (21,915-41,641)	7.8 (5.8-9.8)	51 (32-74)	27,415 (17,310-39,871)	6.8 (4.5-9.5)	13 (6-20)	6,808 (3,135-10,735)	1.7 (0.8-2.6)
Punjab	132 (108-156)	41,090 (33,548-48,366)	18.8 (17.2-20.5)	106 (83-128)	32,771 (25,683-39,595)	15.0 (12.6-17.0)	23 (12-38)	7,204 (3,864-11,894)	3.3 (1.8-5.3)	7 (3-11)	212 (101-331)	1.0 (0.4-1.6)
Tamil Nadu	106 (86-129)	84,587 (68,951-102,758)	13.8 (12.2-15.6)	71 (53-89)	56,630 (42,435-70,904)	9.2 (7.4-11.0)	31 (18-48)	24,657 (14,045-38,009)	4.0 (2.4-6.2)	6 (3-10)	48 (23-76)	0.8 (0.4-1.3)
Maharashtra	112 (91-134)	139,118 (113,462-166,562)	16.7 (14.9-18.5)	76 (59-94)	94,542 (73,575-116,622)	11.4 (9.4-13.2)	29 (17-45)	36,391 (20,730-55,914)	4.4 (2.6-6.6)	11 (5-17)	13,892 (6,647-21,753)	1.7 (0.8-2.5)
Telangana	91 (71-116)	35,364 (27,587-45,295)	15.5 (13.7-17.3)	54 (39-71)	20,843 (15,195-27,595)	9.2 (7.3-10.9)	32 (19-48)	12,314 (7,495-18,667)	5.4 (3.6-7.7)	9 (4-15)	4,741 (2,142-7,911)	1.6 (0.8-2.4)
Kerala	104 (83-127)	36,392 (29,015-44,371)	14.3 (12.2-16.3)	69 (51-89)	24,176 (17,790-30,955)	9.5 (7.4-11.4)	30 (15-51)	10,317 (5,117-17,890)	4.0 (2.1-6.8)	8 (4-13)	2,690 (1,224-4,360)	1.1 (0.5-1.7)
Himachal Pradesh	136 (110-164)	10,383 (8,377-12,510)	18.5 (15.7-21.2)	80 (58-103)	6,072 (4,415-7,833)	10.8 (8.2-13.3)	40 (21-67)	3,040 (1,609-5,138)	5.4 (3.0-8.6)	27 (12-42)	2,066 (919-3,208)	3.7 (1.6-5.5)
Karnataka	131 (106-157)	89,184 (72,283-106,651)	16.6 (14.4-18.6)	66 (48-85)	44,712 (32,862-57,813)	8.3 (6.3-10.3)	57 (36-82)	38671 (24797-55588)	7.2 (4.7-9.9)	14 (7-23)	9,752 (4,493-15,559)	1.8 (0.9-2.8)
Uttarakhand	144 (117-174)	16,989 (13,858-20,537)	18.6 (16.2-20.8)	98 (76-124)	11,639 (8,958-14,665)	12.8 (10.2-15.3)	34 (18-55)	3,979 (2,072-6,492)	4.4 (2.3-6.8)	21 (9-34)	2,514 (1,084-3,974)	2.8 (1.2-4.2)
Gujarat	127 (104-151)	87,811 (71,947-104,322)	18.9 (16.7-20.9)	92 (71-113)	63,922 (49,035-77,985)	13.8 (11.4-16.0)	29 (17-45)	19,915 (11,900-30,888)	4.3 (2.6-6.5)	11 (5-18)	7,919 (3,504-12,768)	1.7 (0.8-2.7)
Haryana	117 (97-139)	34,119 (28,096-40,443)	19.0 (16.9-20.7)	91 (73-110)	26,459 (21,255-32,017)	14.7 (12.6-16.7)	21 (12-34)	6,165 (3,430-9,924)	3.4 (1.9-5.5)	11 (5-18)	3,286 (1,497-5,345)	1.8 (0.8-2.8)
Other small union territories	71 (57-88)	2,688 (2,160-3,358)	13.3 (12.0-14.8)	59 (46-75)	2,257 (1,758-2,836)	11.2 (9.7-12.6)	8 (4-14)	290 (138-521)	1.4 (0.7-2.5)	6 (3-9)	454 (213-705)	1.0 (0.5-1.6)
Sikkim	74 (58-92)	488 (386-607)	14.4 (12.2-16.7)	48 (32-66)	318 (215-438)	9.4 (6.7-12.2)	21 (12-34)	141 (79-228)	4.2 (2.4-6.6)	7 (4-12)	13,289 (4,993-21,988)	1.4 (0.7-2.2)
Delhi	89 (75-103)	17,248 (14,625-20,057)	18.2 (16.4-19.8)	85 (72-99)	16,595 (14,043-19,345)	17.5 (15.8-19.0)	0.4 (0.1-0.9)	77 (28-168)	0.1 (0.0-0.2)	7 (3-11)	1,296 (590-2,108)	1.4 (0.6-2.2)
Goa	91 (69-116)	1,396 (1,059-1,784)	13.3 (11.0-15.5)	79 (58-103)	1,213 (895-1,583)	11.5 (9.2-13.7)	7 (3-15)	114 (46-226)	1.1 (0.5-2.1)	6 (3-10)	95 (45-153)	0.9 (0.4-1.4)

*The states are listed in the increasing order of per capita GDP in 2018-19.

†The sum of deaths attributable to the components of air pollution is more than the estimate for overall air pollution because the population attributable fractions from component risk factors can add up to more than the population attributable fraction for the parent risk factor even if the components are independent.

5. YLL, YLD, and DALY rates attributable to air pollution in the states of India, 2019

States of India*	YLL rate attributable to air pollution (95% UI)	YLD rate attributable to air pollution (95% UI)	DALY rate† attributable to air pollution (95% UI)	YLL rate attributable to ambient particulate matter pollution (95% UI)	YLD rate attributable to ambient particulate matter pollution (95% UI)	DALY rate† attributable to ambient particulate matter pollution (95% UI)	YLL rate attributable to household air pollution (95% UI)	YLD rate attributable to household air pollution (95% UI)	DALY rate† attributable to household air pollution (95% UI)	DALY rate† attributable to ambient ozone pollution‡ (95% UI)
**India**	**3,452 (2,989-3,963)**	**395 (298-496)**	**3,847 (3,350–4,381)**	**2,026 (1,582-2,441)**	**213 (159-273)**	**2,239 (1,768–2,699)**	**1,321 (879-1,831)**	**182 (122-257)**	**1,503 (1,016–2,066)**	220 (108-347)
Bihar	3,486 (2,900-4,093)	365 (279-455)	3,852 (3,235–4,466)	1,719 (1,173-2,323)	155 (104-209)	1,873 (1,291–2,517)	1,701 (1,110-2,371)	211 (140-298)	1,912 (1,281–2,634)	182 (85-285)
Uttar Pradesh	4,611 (3,866-5,455)	391 (299-485)	5,002 (4,227–5,848)	2,886 (2,195-3,587)	220 (165-281)	3,106 (2,387–3,824)	1,583 (984-2,324)	171 (112-248)	1,754 (1,110–2,533)	362 (157-588)
Manipur	2,160 (1,711-2,655)	333 (245-430)	2,492 (2,021–3,024)	1,253 (833-1,706)	169 (107-237)	1,422 (962–1,924)	868 (514-1,313)	163 (103-239)	1,032 (638–1,521)	65 (28-111)
Jharkhand	2,640 (2,205-3,152)	392 (298-491)	3,032 (2,569–3,544)	1,259 (882-1,662)	171 (118-227)	1,429 (1,004–1,870)	1,319 (869-1,834)	221 (149-309)	1,540 (1,043–2,117)	156 (76-242)
Madhya Pradesh	3,954 (3,299-4,660)	378 (288-470)	4,332 (3,671–5,031)	1,844 (1,341-2,357)	168 (120-221)	2,013 (1,474–2,567)	2,012 (1,326-2,717)	209 (141-289)	2,221 (1,505–2,992)	225 (106-356)
Assam	3,308 (2,784-3,896)	342 (254-437)	3,649 (3,095–4,265)	1,294 (886-1,701)	125 (85-171)	1,419 (976–1,842)	1,942 (1,343-2,607)	216 (145-301)	2,158 (1,490–2,909)	131 (59-211)
Meghalaya	1,822 (1,433-2,306)	238 (178-302)	2,059 (1,661–2,550)	727 (493-1,006)	86 (59-117)	813 (557–1,109)	1,056 (718-1,491)	152 (103-211)	1,208 (836–1,670)	68 (31-125)
Jammu & Kashmir and Ladakh	2,903 (2,432-3,451)	321 (243-404)	3,224 (2,720–3,787)	1,912 (1,450-2,360)	207 (151-267)	2,119 (1,624–2,592)	786 (446-1,241)	114 (70-173)	900 (524–1,404)	348 (162-537)
Chhattisgarh	4,030 (3,390-4,665)	432 (321-548)	4,462 (3,822–5,163)	1,695 (1,149-2,279)	158 (107-214)	1,853 (1,251–2,470)	2,239 (1,537-2,989)	274 (188-380)	2,513 (1,769–3,328)	201 (93-317)
West Bengal	3,191 (2,655-3,722)	456 (347-570)	3,647 (3,088–4,183)	1,825 (1,330-2,305)	239 (173-306)	2,064 (1,521–2,574)	1,285 (829-1,820)	217 (144-310)	1,502 (995–2,124)	173 (85-263)
Nagaland	1,959 (1,551-2,461)	263 (194-337)	2,222 (1,801–2,739)	1,015 (696-1,368)	121 (81-165)	1,136 (778–1,513)	919 (551-1,371)	142 (93-204)	1,061 (662–1,553)	41 (17-74)
Odisha	2,795 (2,224-3,441)	402 (298-517)	3,197 (2,612–3,833)	1,211 (834-1,630)	148 (100-203)	1,359 (953–1,808)	1,527 (1,021-2,143)	254 (171-355)	1,781 (1,216–2,441)	109 (46-255)
Rajasthan	4,286 (3,552-5,174)	379 (291-467)	4,665 (3,921–5,536)	2,192 (1,620-2,786)	182 (133-232)	2,374 (1,752–2,991)	1,962 (1,270-2,772)	197 (133-276)	2,159 (1,417–3,004)	301 (114-502)
Tripura	3,116 (2,485-3,812)	409 (306-515)	3,525 (2,887–4,247)	1,620 (1,129-2,211)	190 (129-260)	1,809 (1,280–2,433)	1,389 (900-2,002)	219 (145-310)	1,608 (1,057–2,279)	196 (92-309)
Arunachal Pradesh	1,443 (1,143-1,814)	220 (161-282)	1,664 (1,347–2,042)	519 (338-737)	74 (48-105)	593 (389–823)	894 (596-1,238)	146 (97-205)	1,040 (717–1,411)	48 (21-79)
Mizoram	1,757 (1,366-2,195)	247 (184-317)	2,004 (1,588–2,460)	1,052 (792-1,419)	147 (103-194)	1,199 (915–1,569)	603 (350-913)	100 (63-145)	703 (425–1,039)	150 (60-243)
Andhra Pradesh	2,862 (2,306-3,517)	432 (322-556)	3,294 (2,699–3,957)	1,466 (1,013-1,976)	197 (140-262)	1,663 (1,181–2,197)	1,268 (818-1,829)	235 (153-342)	1,504 (987–2,141)	214 (98-343)
Punjab	3,627 (2,970-4,299)	435 (326-558)	4,062 (3,382–4,751)	2,918 (2,280-3,536)	321 (239-414)	3,239 (2,571–3,846)	644 (345-1,062)	114 (70-175)	758 (423–1,216)	122 (59-208)
Tamil Nadu	2,677 (2,177-3,253)	397 (289-516)	3,075 (2,507–3,666)	1,807 (1,347-2,267)	248 (175-326)	2,055 (1,559–2,564)	796 (458-1,212)	149 (91-225)	945 (554–1,435)	107 (49-178)
Maharashtra	2,746 (2,267-3,267)	397 (299-499)	3,143 (2,638–3,687)	1,909 (1,487-2,357)	252 (188-319)	2,162 (1,706–2,625)	726 (418-1,107)	144 (91-214)	870 (534–1,308)	188 (90-294)
Telangana	2,395 (1,914-3,032)	345 (258-438)	2,741 (2,247–3,394)	1,449 (1,059-1,893)	194 (142-254)	1,643 (1,231–2,102)	847 (531-1,263)	152 (97-219)	999 (654–1,441)	162 (75-265)
Kerala	2,195 (1,750-2,679)	468 (342-605)	2,664 (2,180–3,213)	1,490 (1,093-1,909)	274 (192-365)	1,764 (1,319–2,220)	620 (306-1,067)	194 (111-301)	814 (442–1,340)	121 (55-197)
Himachal Pradesh	3,165 (2,596-3,831)	431 (322-547)	3,596 (2,997–4,288)	1,916 (1,370-2,472)	240 (169-321)	2,156 (1,564–2,750)	966 (520-1,604)	191 (117-289)	1,157 (668–1,883)	459 (204-719)
Karnataka	3,309 (2,711-3,953)	387 (287-496)	3,695 (3,067–4,389)	1,712 (1,251-2,226)	189 (133-254)	1,901 (1,402–2,446)	1,449 (940-2,042)	198 (127-283)	1,647 (1,085–2,292)	247 (114-394)
Uttarakhand	3,732 (3,092-4,459)	413 (312-521)	4,145 (3,492–4,875)	2,627 (2,037-3,296)	272 (197-353)	2,899 (2,259–3,608)	884 (471-1,425)	141 (87-211)	1,025 (571–1,607)	405 (177-642)
Gujarat	3,578 (2,964-4,224)	394 (300-492)	3,973 (3,323–4,637)	2,632 (2,032-3,190)	267 (202-339)	2,899 (2,292–3,472)	841 (513-1,283)	127 (81-186)	968 (609–1,441)	210 (95-338)
Haryana	3,320 (2,810-3,895)	430 (330-540)	3,750 (3,211–4,322)	2,608 (2,122-3,138)	308 (236-380)	2,917 (2,394–3,485)	620 (345-994)	122 (77-179)	742 (437–1,156)	200 (92-328)
Other small union territories	1,798 (1,445-2,250)	300 (223-389)	2,098 (1,709–2,562)	1,535 (1,188-1,939)	243 (179-315)	1,777 (1,403–2,198)	198 (93-357)	58 (31-93)	255 (133–434)	99 (48-158)
Sikkim	1,797 (1,404-2,252)	336 (245-432)	2,133 (1,706–2,603)	1,217 (824-1,670)	210 (140-284)	1,426 (985–1,920)	505 (280-808)	126 (78-191)	631 (365–976)	123 (59-194)
Delhi	2,417 (2,048-2,806)	366 (279-458)	2,783 (2,391–3,192)	2,353 (1,997-2,739)	358 (273-446)	2,711 (2,337–3,108)	11 (4-24)	8 (3-16)	19 (9–37)	118 (55-193)
Goa	2,023 (1,526-2,603)	385 (278-505)	2,408 (1,870–3,020)	1,791 (1,320-2,339)	325 (231-433)	2,116 (1,616–2,701)	159 (63-316)	60 (31-104)	218 (101–407)	102 (48-166)

*The states are listed in the increasing order of per capita GDP in 2018-19.
†The sum of DALYs attributable to the components of air pollution is more than the estimate for overall air pollution because the population attributable fractions from component risk factors can add up to more than the population attributable fraction for the parent risk factor even if the components are independent.
‡There are no YLDs attributed to ozone in GBD, so all DALYs from ozone are due to YLLs.

6. Economic loss due to premature deaths and morbidity attributable to air pollution in the states of India, 2019

States of India*	Economic loss (US$, millions) attributable to			
	Air pollution	Ambient particulate matter pollution	Household air pollution	Ambient ozone pollution
**India**	36,803.8 (27,368.6-47,710.3)	22,788.6 (15,936.6-30,704.6)	13,300.0 (7,861.1-20,370.5)	1,419.6 (624.4-2,375.1)
Bihar	1,552.8 (1,153.4-2,022.5)	751.1 (474.6-1,075.6)	781.3 (478.8-1,169.3)	55.8 (25.6-91.1)
Uttar Pradesh	5,130.3 (3,816.0-6,616.1)	3,188.4 (2,241.7-4,296.0)	1,829.6 (1,061.3-2,816.1)	286.2 (120.1-482.4)
Manipur	40.5 (28.8-54.7)	23.0 (14.3-33.8)	17.1 (9.8-26.8)	0.7 (0.3-1.2)
Jharkhand	543.3 (398.2-7,14.9)	257.6 (166.6-367.2)	278.1 (170.6-413.9)	19.1 (8.9-31.2)
Madhya Pradesh	1,970.5 (1,479.8-2,541.8)	926.3 (626.3-1,267.7)	1,012.5 (632.3-1,470.8)	72.7 (33.4-118.3)
Assam	657.0 (483.1-864.7)	257.6 (165.4-365.7)	390.3 (246.2-571.1)	16.4 (7.2-27.5)
Meghalaya	39.2 (27.0-54.7)	15.6 (9.6-23.4)	23.1 (13.9-35.3)	0.9 (0.4-1.8)
Jammu & Kashmir and Ladakh	252.2 (188.1-326.7)	168.5 (118.4-224.4)	72.5 (39.6-119.2)	18.9 (8.6-30.6)
Chhattisgarh	690.0 (512.1-893.8)	287.2 (182.1-409.8)	392.6 (248.2-566.9)	21.1 (9.6-34.7)
West Bengal	2,125.3 (1,622.8-2,676.8)	1,204.0 (838.8-1,601.1)	890.6 (555.2-1,323.3)	65.1 (30.9-105.3)
Nagaland	33.6 (23.0-46.9)	17.3 (10.7-25.6)	16.0 (9.0-25.7)	0.4 (0.2-0.8)
Odisha	806.6 (573.6-1,088.1)	341.3 (216.8-496.8)	455.7 (281.2-678.2)	18.5 (7.6-43.9)
Rajasthan	2,294.3 (1,673.8-2,996.2)	1,178.5 (799.2-1,617.6)	1,069.3 (639.5-1,613.5)	105.2 (39.7-178.2)
Tripura	91.1 (66.1-121.0)	47.4 (30.7-67.6)	41.9 (25.3-63.6)	3.3 (1.5-5.5)
Arunachal Pradesh	26.0 (17.6-36.5)	9.5 (5.7-14.4)	16.2 (9.7-24.8)	0.5 (0.2-0.8)
Mizoram	22.4 (15.3-31.3)	13.7 (8.8-20.0)	8.0 (4.3-12.8)	1.1 (0.4-1.9)
Andhra Pradesh	1,349.5 (968.9-1,817.8)	692.2 (455.4-985.3)	623.7 (375.6-954.7)	56.4 (25.0-94.2)
Punjab	1,148.9 (862.2-1,474.4)	916.6 (666.3-1,191.0)	219.7 (116.9-367.6)	23.7 (10.9-41.7)
Tamil Nadu	2,529.1 (1,856.6-3,310.4)	1,693.3 (1,174.3-2,283.7)	796.6 (443.9-1,263.3)	56.5 (25.2-96.2)
Maharashtra	3,975.4 (3,003.6-5,079.6)	2,765.8 (1,992.1-3,632.4)	1,119.3 (636.3-1,753.7)	152.6 (70.8-248.4)
Telangana	1,115.9 (792.7-1,508.2)	681.1 (461.8-948.0)	409.3 (239.5-636.3)	41.6 (18.9-71.5)
Kerala	1,090.5 (808.0-1,420.7)	722.7 (496.2-973.3)	346.0 (177.0-588.5)	30.6 (13.9-52.0)
Himachal Pradesh	253.8 (187.8-331.4)	154.8 (104.3-213.4)	85.6 (45.9-143.3)	21.8 (9.6-35.7)
Karnataka	2,680.7 (2,006.0-3,459.2)	1,406.4 (960.8-1,937.8)	1,204.7 (736.6-1,788.2)	116.5 (53.2-191.4)
Uttarakhand	526.6 (392.8-682.7)	373.5 (266.2-503.3)	133.9 (70.1-220.0)	35.1 (14.8-57.1)
Gujarat	2,859.6 (2,157.9-3,667.0)	2,100.7 (1,526.0-2,749.9)	706.9 (402.9-1,123.9)	103.1 (45.5-170.9)
Haryana	1,566.3 (1,187.9-2,009.1)	1,221.7 (905.2-1,591.0)	318.8 (173.0-523.6)	56.5 (25.0-94.2)
Other small union territories	120.3 (85.5-163.7)	102.5 (71.4-141.0)	15.4 (7.4-27.9)	3.5 (1.6-5.8)
Sikkim	25.5 (18.0-34.7)	17.5 (11.2-25.2)	7.5 (4.1-12.4)	0.9 (0.4-1.4)
Delhi	1,206.5 (906.2-1,554.8)	1,182.0 (887.3-1,523.4)	9.9 (3.7-21.2)	32.8 (14.3-56.1)
Goa	79.9 (55.5-110.0)	70.8 (48.4-99.0)	7.6 (3.3-14.8)	2.0 (0.9-3.4)

*The states are listed in the increasing order of per capita GDP in 2018-19.

7. Economic loss due to premature deaths and morbidity attributable to air pollution as a percentage of state GDP in India, 2019

	Economic loss as a percentage of state GDP due to									
	Air pollution			Ambient particulate matter pollution			Household air pollution			Ambient ozone pollution†
States of India*	Premature deaths	Morbidity	Total	Premature deaths	Morbidity	Total	Premature deaths	Morbidity	Total	Total
**India**	1.06 (0.79-1.38)	0.30 (0.22-0.38)	1.36 (1.01-1.76)	0.66 (0.46-0.90)	0.17 (0.12-0.23)	0.84 (0.59-1.13)	0.37 (0.21-0.57)	0.12 (0.08-0.18)	0.49 (0.29-0.75)	0.05 (0.02-0.09)
Bihar	1.58 (1.17-2.06)	0.37 (0.28-0.47)	1.95 (1.45-2.54)	0.78 (0.49-1.13)	0.16 (0.11-0.22)	0.94 (0.60-1.35)	0.77 (0.46-1.16)	0.21 (0.14-0.30)	0.98 (0.60-1.47)	0.07 (0.03-0.11)
Uttar Pradesh	1.78 (1.32-2.31)	0.37 (0.28-0.46)	2.15 (1.60-2.77)	1.12 (0.79-1.53)	0.21 (0.15-0.27)	1.34 (0.94-1.80)	0.61 (0.35-0.95)	0.16 (0.10-0.23)	0.77 (0.44-1.18)	0.12 (0.05-0.20)
Manipur	0.79 (0.56-1.08)	0.29 (0.21-0.37)	1.08 (0.77-1.46)	0.46 (0.29-0.69)	0.15 (0.09-0.21)	0.61 (0.38-0.90)	0.32 (0.18-0.51)	0.14 (0.08-0.20)	0.46 (0.26-0.72)	0.02 (0.01-0.03)
Jharkhand	0.93 (0.68-1.23)	0.31 (0.23-0.39)	1.24 (0.91-1.63)	0.45 (0.28-0.65)	0.14 (0.09-0.19)	0.59 (0.38-0.83)	0.46 (0.28-0.70)	0.17 (0.11-0.24)	0.63 (0.39-0.94)	0.04 (0.02-0.07)
Madhya Pradesh	1.39 (1.05-1.81)	0.31 (0.23-0.39)	1.70 (1.28-2.20)	0.66 (0.44-0.91)	0.14 (0.10-0.19)	0.80 (0.54-1.10)	0.71 (0.44-1.04)	0.17 (0.11-0.23)	0.87 (0.55-1.27)	0.06 (0.03-0.10)
Assam	1.14 (0.84-1.51)	0.28 (0.20-0.36)	1.42 (1.04-1.87)	0.45 (0.29-0.64)	0.11 (0.07-0.15)	0.56 (0.36-0.79)	0.67 (0.42-0.99)	0.17 (0.11-0.24)	0.84 (0.53-1.23)	0.04 (0.02-0.06)
Meghalaya	0.61 (0.41-0.87)	0.19 (0.14-0.24)	0.80 (0.55-1.11)	0.25 (0.15-0.38)	0.07 (0.05-0.10)	0.32 (0.20-0.47)	0.35 (0.21-0.55)	0.12 (0.08-0.17)	0.47 (0.28-0.72)	0.02 (0.01-0.04)
Jammu & Kashmir and Ladakh	0.91 (0.68-1.18)	0.23 (0.17-0.30)	1.14 (0.85-1.48)	0.61 (0.43-0.81)	0.15 (0.11-0.20)	0.76 (0.54-1.02)	0.25 (0.13-0.42)	0.08 (0.05-0.12)	0.33 (0.18-0.54)	0.09 (0.04-0.14)
Chhattisgarh	1.23 (0.92-1.60)	0.32 (0.23-0.41)	1.55 (1.15-2.01)	0.52 (0.33-0.75)	0.12 (0.08-0.17)	0.64 (0.41-0.92)	0.69 (0.43-1.00)	0.20 (0.13-0.27)	0.88 (0.56-1.27)	0.05 (0.02-0.08)
West Bengal	0.95 (0.73-1.20)	0.31 (0.23-0.39)	1.26 (0.96-1.59)	0.55 (0.38-0.73)	0.16 (0.12-0.22)	0.71 (0.50-0.95)	0.39 (0.24-0.58)	0.14 (0.09-0.21)	0.53 (0.33-0.79)	0.04 (0.02-0.06)
Nagaland	0.66 (0.44-0.94)	0.20 (0.15-0.26)	0.86 (0.59-1.20)	0.35 (0.21-0.52)	0.10 (0.06-0.13)	0.44 (0.27-0.66)	0.30 (0.16-0.50)	0.11 (0.07-0.15)	0.41 (0.23-0.66)	0.01 (0.00-0.02)
Odisha	0.86 (0.61-1.17)	0.28 (0.20-0.36)	1.14 (0.81-1.53)	0.37 (0.23-0.55)	0.11 (0.07-0.15)	0.48 (0.31-0.70)	0.47 (0.28-0.71)	0.17 (0.11-0.24)	0.64 (0.40-0.96)	0.03 (0.01-0.06)
Rajasthan	1.41 (1.02-1.86)	0.29 (0.22-0.37)	1.70 (1.24-2.22)	0.73 (0.49-1.01)	0.14 (0.10-0.19)	0.87 (0.59-1.20)	0.65 (0.38-0.99)	0.15 (0.10-0.21)	0.79 (0.47-1.20)	0.08 (0.03-0.13)
Tripura	0.97 (0.70-1.30)	0.30 (0.22-0.38)	1.26 (0.92-1.68)	0.51 (0.33-0.74)	0.14 (0.10-0.20)	0.66 (0.43-0.94)	0.43 (0.25-0.66)	0.15 (0.10-0.22)	0.58 (0.35-0.88)	0.05 (0.02-0.08)
Arunachal Pradesh	0.54 (0.36-0.78)	0.20 (0.14-0.26)	0.74 (0.50-1.04)	0.20 (0.12-0.31)	0.07 (0.04-0.10)	0.27 (0.16-0.41)	0.33 (0.19-0.52)	0.13 (0.08-0.18)	0.46 (0.28-0.70)	0.01 (0.01-0.02)
Mizoram	0.52 (0.35-0.75)	0.18 (0.13-0.23)	0.70 (0.48-0.98)	0.32 (0.20-0.48)	0.11 (0.08-0.15)	0.43 (0.28-0.63)	0.18 (0.09-0.30)	0.07 (0.04-0.10)	0.25 (0.14-0.40)	0.03 (0.01-0.06)
Andhra Pradesh	0.82 (0.58-1.11)	0.28 (0.20-0.36)	1.09 (0.79-1.47)	0.43 (0.28-0.62)	0.13 (0.09-0.18)	0.56 (0.37-0.80)	0.36 (0.21-0.56)	0.15 (0.09-0.21)	0.51 (0.30-0.77)	0.05 (0.02-0.08)
Punjab	1.22 (0.92-1.56)	0.30 (0.22-0.40)	1.52 (1.14-1.96)	0.99 (0.72-1.28)	0.23 (0.17-0.30)	1.22 (0.88-1.58)	0.22 (0.11-0.37)	0.07 (0.04-0.12)	0.29 (0.16-0.49)	0.03 (0.01-0.06)
Tamil Nadu	0.79 (0.59-1.03)	0.27 (0.19-0.36)	1.06 (0.78-1.39)	0.54 (0.37-0.73)	0.17 (0.12-0.23)	0.71 (0.49-0.96)	0.24 (0.13-0.38)	0.10 (0.06-0.15)	0.33 (0.19-0.53)	0.02 (0.01-0.04)
Maharashtra	0.80 (0.61-1.02)	0.26 (0.19-0.33)	1.06 (0.80-1.35)	0.56 (0.41-0.74)	0.17 (0.12-0.22)	0.73 (0.53-0.96)	0.21 (0.12-0.33)	0.09 (0.05-0.13)	0.30 (0.17-0.47)	0.04 (0.02-0.07)
Telangana	0.68 (0.48-0.94)	0.22 (0.16-0.29)	0.91 (0.64-1.22)	0.42 (0.28-0.60)	0.13 (0.09-0.17)	0.55 (0.37-0.77)	0.24 (0.14-0.38)	0.09 (0.06-0.14)	0.33 (0.19-0.52)	0.03 (0.02-0.06)
Kerala	0.66 (0.50-0.86)	0.31 (0.23-0.41)	0.98 (0.72-1.27)	0.46 (0.32-0.61)	0.19 (0.13-0.26)	0.65 (0.44-0.87)	0.19 (0.09-0.33)	0.12 (0.07-0.20)	0.31 (0.16-0.53)	0.03 (0.01-0.05)
Himachal Pradesh	0.88 (0.65-1.15)	0.28 (0.21-0.37)	1.16 (0.86-1.51)	0.54 (0.36-0.75)	0.16 (0.11-0.22)	0.71 (0.48-0.97)	0.27 (0.14-0.47)	0.12 (0.07-0.19)	0.39 (0.21-0.65)	0.10 (0.04-0.16)
Karnataka	0.96 (0.73-1.24)	0.26 (0.19-0.34)	1.22 (0.91-1.58)	0.51 (0.35-0.70)	0.13 (0.09-0.18)	0.64 (0.44-0.88)	0.42 (0.26-0.63)	0.13 (0.08-0.18)	0.55 (0.34-0.81)	0.05 (0.02-0.09)
Uttarakhand	1.17 (0.88-1.53)	0.32 (0.24-0.41)	1.50 (1.12-1.94)	0.84 (0.60-1.14)	0.22 (0.16-0.29)	1.06 (0.76-1.43)	0.28 (0.14-0.47)	0.10 (0.06-0.16)	0.38 (0.20-0.63)	0.10 (0.04-0.16)
Gujarat	1.07 (0.80-1.37)	0.27 (0.20-0.34)	1.33 (1.00-1.71)	0.79 (0.57-1.04)	0.19 (0.14-0.24)	0.98 (0.71-1.28)	0.25 (0.14-0.40)	0.08 (0.05-0.12)	0.33 (0.19-0.52)	0.05 (0.02-0.08)
Haryana	1.17 (0.89-1.50)	0.33 (0.25-0.41)	1.49 (1.13-1.91)	0.92 (0.68-1.21)	0.24 (0.18-0.30)	1.16 (0.86-1.52)	0.22 (0.11-0.37)	0.09 (0.05-0.13)	0.30 (0.16-0.50)	0.05 (0.02-0.09)
Other small union territories	0.56 (0.40-0.77)	0.23 (0.16-0.30)	0.79 (0.56-1.07)	0.48 (0.33-0.67)	0.19 (0.13-0.25)	0.67 (0.47-0.92)	0.06 (0.03-0.12)	0.04 (0.02-0.07)	0.10 (0.05-0.18)	0.02 (0.01-0.04)
Sikkim	0.46 (0.32-0.63)	0.21 (0.15-0.27)	0.67 (0.47-0.91)	0.32 (0.20-0.47)	0.14 (0.09-0.19)	0.46 (0.29-0.66)	0.12 (0.06-0.21)	0.07 (0.04-0.11)	0.20 (0.11-0.32)	0.02 (0.01-0.04)
Delhi	0.80 (0.60-1.03)	0.28 (0.21-0.36)	1.08 (0.81-1.39)	0.78 (0.59-1.01)	0.28 (0.21-0.35)	1.06 (0.80-1.37)	0.00 (0.00-0.01)	0.01 (0.00-0.01)	0.01 (0.00-0.02)	0.03 (0.01-0.05)
Goa	0.49 (0.34-0.68)	0.24 (0.17-0.32)	0.72 (0.50-1.00)	0.44 (0.30-0.62)	0.20 (0.14-0.28)	0.64 (0.44-0.90)	0.04 (0.01-0.08)	0.03 (0.02-0.06)	0.07 (0.03-0.13)	0.02 (0.01-0.03)

GDP is Gross Domestic Product.
*The states are listed in the increasing order of per capita GDP in 2018-19.
†There are no YLDs attributed to ozone in GBD, so there is no economic loss attributable to morbidity. All of the economic loss from ozone is due to premature deaths.

8. Air pollution control initiatives in India

Air pollution control initiatives	Description
**National Programme on Climate Change and Human Health**[1]	National Programme on Climate Change and Human Health was launched in February 2019 by the National Centre for Disease Control, Ministry of Health and Family Welfare to combat factors, including air pollution, that bring about an increase in climate sensitive illnesses, develop strategies to adapt to climate change consequences, and to build a climate resilient health system in India. Major air pollution related activities undertaken are initiation of sentinel surveillance of acute respiratory illnesses from emergencies of major hospitals across India to visualize their trend in backdrop of air pollution levels of these cities; development of health adaptation plan on air pollution for states; development of content and running campaigns for disseminating health messages on air pollution and its impacts for health professionals and the community; and fostering partnership with relevant ministries, technical organisations, and development partners to identify evidence based actions to protect from air pollution. This programme is being implemented by all state health departments and in the past one year several awareness generation activities have been undertaken by state governments.
**National Clean Air Programme**[2,3]	Clean Air for Delhi Campaign was launched for ten days in late 2018 deploying 52 teams in Delhi to monitor, report polluting activities and ensure quick action. This campaign then evolved to the National Clean Air Programme (NCAP), which is a mid-term five-year action plan launched in 2019. The long-term goal of the NCAP is to meet the prescribed annual average ambient air quality standards at all locations in the country in a stipulated timeframe. The national level target of this programme is 20%–30% reduction of $PM_{2.5}$ and PM_{10} concentration by 2024 in 102 cities. The objectives of this programme are to ensure stringent implementation of mitigation measures for prevention, control and abatement of air pollution; augment and evolve effective and proficient ambient air quality monitoring network across the country for ensuring a comprehensive and reliable database; augment public awareness and capacity-building measures encompassing data dissemination and public outreach programme for inclusive public participation and for ensuring trained manpower and infrastructure on air pollution.
**National Air Quality Monitoring Programme**[3, 4-6]	A nation-wide programme of ambient air quality monitoring, National Ambient Air Quality Monitoring programme was initiated by Central Pollution Control Board in the year 1984 with 7 stations which gained momentum only recently and got renamed as National Air Monitoring Programme. The network of this programme consists of 779 operating stations covering 339 cities/towns in 29 states and 6 union territories of the country. The objectives of this programme are to determine the status and trends of ambient air quality; to ascertain whether the prescribed ambient air quality standards are violated; to identify non-attainment cities; to obtain the knowledge and understanding necessary for developing preventive and corrective measures; and to understand the natural cleansing process undergoing in the environment through pollution dilution, dispersion, wind-based movement, dry deposition, precipitation, and chemical transformation of the pollutants generated.
**Pradhan Mantri Ujjwala Yojana**[7-10]	Launched in May 2016 by the Prime Minister to provide free 50 million LPG connections to BPL households by 2020. This target was achieved before time towards the end of 2018 and the target was renewed to 80 million of households which was achieved by September 2019.<br><br>As off shoots of Ujjwala Yojana, there were two separate initiatives launched to encourage the use of clean cooking fuel.<br>o Pradhan Mantri LPG panchayats: Launched in March 2018 is a peer learning platforms, Pradhan Mantri LPG panchayats are providing support, catalysing behaviour changes in Ujjawala beneficiaries and also encouraging safe and sustainable use of LPG. As many as 87,876 LPG panchayats have been conducted across the country.<br>o Ujwala Didi: A corporate social responsibility handholding initiative launched in 2019, aims at creating a force of 10,000 grass root educators who can take the three messages till the last mile, viz. (i) clean cooking fuel is to be universally available, (ii) clean cooking fuel is affordable and (iii) LPG is safe to use and insured. Ujjwala Didis will facilitate refill, address any fear around LPG safety, help in resolving any grievances, and facilitate new connections.
**Unnat Chulha Abhiyan**[11-13]	National Biomass Cookstove Programme was launched in 2009 by Ministry of New and Renewable Energy. The primary aim was to enhance the availability of clean and efficient energy for the energy deficient and poorer sections of the country. This initiative recognised that cook stove technology has improved considerably in the recent years and the efforts need to be continued further. This programme was relaunched as Unnat Chulha Abhiyan in 2014 by Ministry of New and Renewable Energy. This programme aimed at promotion of improved biomass cook stove in the country for providing a clean cooking energy solution with a view to reduce consumption of fuel wood with higher efficiency and lower emissions. The improved cook stoves were distributed to 36,940 types of families and 849 communities.
**The Air (Prevention and Control of Pollution) Act 1981, amended 1987**[14]	This act was started in 1981 for the prevention, control and abatement of air pollution in India. It was amended finally in 1987.<br>In 2016, the Central Pollution Control Board issued a comprehensive set of directions under section 18 (1) (b) of the Air (Prevention and Control of Pollution) Act, 1981 for implementation of 42 points

	measures to mitigate air pollution in major cities in India. These are action points to counter air pollution including control and mitigation measures related to vehicular emissions, re-suspension of road dust and other fugitive emissions, bio-mass/municipal solid waste burning, industrial pollution, and construction and demolition activities.
**100 Smart Cities Mission**[15]	This mission was launched on June 2015 and managed by the Union Ministry of Housing and Urban Affairs. This urban mission aims to develop 100 cities across the country that are citizen-friendly and sustainable. Specific initiatives are the traffic planning, congestion pricing and increased pedestrian areas in city centers; increased fuel taxes and parking fees; improvements to public transportation systems; removal of sub-standard vehicles that fail emissions testing; upgrading of diesel exhaust controls; increase the usage of electric vehicles; reduce reliance on diesel generator sets by improving power supplies; require systems which use low-sulphur fuels in generators and boilers; upgrade municipal solid waste collection and disposal to reduce street side waste burning.
**Environment Pollution (Prevention and Control) Authority (EPCA)**[3]	Environment Pollution (Prevention and Control) Authority (EPCA) was constituted under Section 3(3) of Environment (Protection) Act, 1986, in 1998 to assess matters pertaining to environmental pollution in the National Capital Region (NCR).
**Green Good Deeds Initiative (National Green Corps programme)**[16-18]	Popularly known as 'the programme of eco clubs', this programme which was launched in 2001-2002 for creating environmental awareness among children, which has now spread across to one lakh twenty thousand schools. This programme aimed at building cadres of young students working towards environmental conservation for more secure and sustainable world. Students are actively involved in various environment protection and conservation activities, the interactions with the environment including the issues related to the air pollution. This has now been included as a component and launched as a initiative called Green Good Deeds Initiative in December 2019. The objective of this initiative is to promote environmental awareness among all sections of the society and to mobilize people's participation for conservation of environment.
**Doctors for Clean Air**[19]	'Doctors for Clean Air' campaign was launched in December 2018 by the Lung Care Foundation in partnership with Health Care Without Harm, bringing together more than 40 senior doctors representing every state of the country who have pledged to be champions of clean air by highlighting the health ill-effects of air pollution and promoting viable solutions. It is a network of 150,000 specialist doctors from twelve leading National Medical Associations of India who have committed themselves to this cause and will work for clean air for all in India. The key objective of this campaign are creating awareness among general public about the serious ill-effects of air pollution and the damage to their health and future generations due to breathing dirty air, sensitizing citizens and policy makers regarding the threat to mankind due to air pollution and make then recognize air pollution as a public health emergency; and influencing union and state policy makers and administrators towards formulation and implementation of urgent and stricter air pollution control policies and laws.
**Construction & Demolition Waste Management Rules, 2016**[20, 21]	The Government has notified Construction & Demolition Waste Management Rules, 2016 with the goal of separating, recovering, recycling and reusing waste generated through construction and demolition. In 2018, notification of dust mitigation measures in construction and demolition activities were introduced to be strictly followed.
**Sameer App**[22]	Sameer application was launched in 2016. Air quality information is available to public along with provision for registering complaints against air polluting activities through this android appilcation.
**Graded Response Action Plan (GRAP) - Delhi and surrounds**[22,23]	The Government notified a Graded Response Action Plan for Delhi and the NCR in 2017, which comprised of graded measures for each pollution source framed according to Air Quality Index categories. These interventions included prohibition on entry of trucks into Delhi; ban on construction activities; introduction of odd and even scheme for private vehicles; shutting of schools; closure of brick kilns; hot mix plants and stone crushers; shutting down of Badarpur power plant; ban on diesel generator sets; garbage burning in landfills and using visibly polluting vehicles; conversion of 2,789 brick kilns in NCR to zig-zag technology and install vapour recovery systems. Moreover, the plan asks for better traffic management and mentioned a need to improve the frequency of the metro while introducing more buses into the system. 46 teams were deployed to monitor air pollution levels in Delhi and other NCR places from October 2019.
**SATAT Inititaive**[24]	The initiative launched in October 2018 is aimed at providing a 'Sustainable Alternative Towards Affordable Transportation' as a developmental effort that would benefit both vehicle-users as well as farmers and entrepreneurs. This initiative holds great promise for efficient municipal solid waste management and in tackling the problem of polluted urban air due to farm stubble-burning and carbon emissions.
**National Biofuel Policy**[25]	The policy aims to increase usage of biofuels in the energy and transportation sectors of the country during the coming decade. It also aims to utilize, develop and promote domestic feedstock and its utilization for production of biofuels. Simultaneously, the policy will also encourage the application of advance technologies for generation of biofuels, to enable availability of biofuels, and increasing blending percentages in petrol and diesel.<br><br>Currently the ethanol blending percentage in petrol is around 2.0% and biodiesel blending percentage in diesel is less than 0.1%. An indicative target of 20% blending of ethanol in petrol and 5% blending of biodiesel in diesel is proposed by 2030.
**Promotion of agricultural mechanization for in-situ management of crop residue in**	160.8 million USD was released for the period from 2018-19 to 2019-20 by the Government of India to tackle air pollution and to subsidize machinery required for in-situ management of crop residue in the States of Punjab, Haryana, Uttar Pradesh and NCR of Delhi. Within one year of its

**the state of Punjab, Haryana, Uttar Pradesh & NCR of Delhi**[26]	implementation the happy seeder/zero tillage technology was adopted in 0.8 million hectares of land in the north-western states of India. Under this scheme, financial assistance of 50% of the cost is provided to the farmers for purchase of in-situ crop residue management machines on individual ownership basis.
**Green fire crackers**[27]	These are environmental friendly fire crackers launched by government in October 2019 to reduce air pollution emitted from fire crackers used in Diwali celebration in India.
**Upgrading BS IV to BS VI by April 2020**[28, 29]	This was intially announced in 2016, and introduced in 2019 by the Minstry of Environment, Forest and Climate Change. This plan aims to completely shift from BS IV to BS VI vehicular emission norms by April 2020. US$ 8,376 million were spent on switching over to BS VI fuels.
**National Electric Mobility Mission Plan 2020**[30]	This plan provides the vision and the roadmap for the faster adoption of electric vehicles and their manufacturing in the country. This plan has been designed to enhance national fuel security, to provide affordable and environmentally friendly transportation, and to enable the Indian automotive industry to achieve global manufacturing leadership.
**National E-Mobility Programme**[31]	This programme aims to provide an momentum to the entire e-mobility ecosystem including vehicle manufacturers, charging infrastructure companies, fleet operators, and service providers to save fuel. By 2030, it aims to convert 30% of the transport as electric vehicles.

References

1. National Centre for Disease Control, Ministry of Health and Family Welfare. NCDC Newsletter 2019; 8.
2. Press Information Bureau, Ministry of Environment Forest and Climate Change, Government of India. Government launches National Clean Air Programme (NCAP). https://pib.gov.in/pressreleaseiframepage.aspx?prid=1559384 (accessed Dec 11, 2020).
3. Ministry of Environment, Forest and Climate Change, Government of India. NCAP-National Clean Air Programme. New Delhi, 2019 http://moef.gov.in/wp-content/uploads/2019/05/NCAP_Report.pdf (accessed Dec 11, 2020).
4. Rangarajan R. Air quality monitoring regime in India-an overview. Pollution monitoring series: briefing note 1. Centre for Development Finance, 2009 http://www.indiaenvironmentportal.org.in/files/Air%20pollution%20note_final.pdf.
5. Press Information Bureau, Ministry of Environment, Forest and Climate Change, Government of India. Revised national ambient air quality standards, 2009. http://moef.gov.in/wp-content/uploads/2019/10/Press-Note-on-RNAAQS_1.pdf (accessed Dec 11, 2020).
6. Central Pollution Control Board, Ministry of Environment, Forest and Climate Change, Government of India. National Air Quality Monitoring Programme. 2017 http://cpcb.nic.in/about-namp/ (accessed Dec 11, 2020).
7. Press Information Bureau, Ministry of Petroleum & Natural Gas, Government of India. LPG connection to every household. 2018 https://pib.gov.in/Pressreleaseshare.aspx?PRID=1525181 (accessed Dec 11, 2020).
8. Ministry of Petroleum & Natural Gas, Government of India. Pradhan Mantri Ujjwala Yojana. 2018. http://petroleum.nic.in/sites/default/files/PMUY_SchemeE.pdf (accessed Dec 11, 2020).
9. Press Information Bureau, Cabinet Committee on Economic Affairs, Government of India. Cabinet approves enhancement of target under Pradhan Mantri Ujjwala Yojana. https://pib.gov.in/PressReleseDetail.aspx?PRID=1519602 (accessed Dec 11, 2020).
10. Press Information Bureau, Ministry of Petroleum & Natural Gas, Government of India. Release of 8 crore PMUY connection by Hon'ble Prime Minister. https://pib.gov.in/PressReleaseIframePage.aspx?PRID=1584346#:~:text=Release%20of%208%20croreth%20PMUY%20connection%20by%20Hon'ble%20Prime%20Minister&text=Hon'ble%20Prime%20Minister%20Shri,on%207th%20September%2C%202019 (accessed Dec 11, 2020).
11. Venkataraman C, Sagar AD, Habib G, Lam N, Smith KR. The Indian National Initiative for Advanced Biomass Cookstoves: the benefits of clean combustion. Energy Sustain Dev 2010;14: 63–72.
12. Press Information Bureau, Ministry of New and Renewable Energy, Government of India. Government making efforts to reduce dependence on traditional biomass cooking (22/03/2019). pib.gov.in/Pressreleaseshare.aspx?PRID=1525934 (accessed Dec 11, 2020).
13. Ministry of New and Renewable Energy, Government of India. National Biomass CookStove Programme (NBCP). http://164.100.94.214/national-biomass-cookstoves-programme (accessed Dec 11, 2020).
14. Centre of Pollution Control Board, Ministry of Environment, Forest and Climate Change, Government of India. The Air (Prevention and Control of Pollution) Act, 1981. https://cpcb.nic.in/displaypdf.php?id=aG9tZS9haXItcG9sbHV0aW9uL05vLTE0LTE5ODEucGRm (accessed Dec 11, 2020).

15. Ministry of Housing and Urban Affairs, Government of India. Smart Cities Mission. 2017 http://smartcities.gov.in/content/innerpage/smart-city-features.php (accessed Dec 11, 2020).
16. Ministry of Environment, Forest and Climate Change. National Green Corps Monitoring System. http://ngc.nic.in/Home.aspx (accessed Dec 11, 2020).
17. Environmental Planning and Coordination Organisation. National Green Corps. http://www.epco.in/epco_education_ngc.php (accessed Dec 11, 2020).
18. Press Information Bureau, Ministry of Environment, Forest and Climate Change, Government of India. 'Green Good Deeds' initiative to promote environmental awareness and to mobilize people's participation for conservation of environment. https://pib.gov.in/Pressreleaseshare.aspx?PRID=1595520 (accessed Dec 11, 2020).
19. Lung Care Foundation and Health Care Without Harm. Doctors for Clean Air | A Public Health Emergency (accessed Dec 11, 2020).
20. Press Information Bureau, Ministry of Environment, Forest and Climate Change, Government of India. Environment ministry notifies construction and demolition waste management rules for the first time. https://archive.pib.gov.in/archive2/PrintRelease.aspx (accessed Dec 11, 2020).
21. Press Information Bureau, Ministry of Environment, Forest and Climate Change, Government of India. Measures to tackle dust pollution. 2018 https://pib.gov.in/Pressreleaseshare.aspx?PRID=1541778 (accessed Dec 11, 2020).
22. Press Information Bureau, Ministry of Environment Forest and Climate Change, Government of India. Various Initiatives undertaken by Government for mitigation of Air Pollution. 2019 https://pib.gov.in/PressReleasePage.aspx?PRID=1593056#:~:text=The%20Central%20Government%20has%20launched,reduction%20in%20PM10%20and (accessed Dec 11, 2020).
23. Press Information Bureau, Ministry of Environment, Forest and Climate Change, Government of India. Graded Action Plan to reduce urban air pollution. 2017 https://pib.gov.in/PressReleaseIframePage.aspx?PRID=1484959 (accessed Dec 11, 2020).
24. Press Information Bureau, Ministry of Petroleum and Natural Gas, Government of India. Petroleum Minister launches SATAT initiative to promote compressed bio-gas as an alternative, green transport fuel. pib.nic.in/Pressreleaseshare.aspx?PRID=1548031 (accessed Dec 11, 2020).
25. Ministry of Petroleum & Natural Gas, Government of India. National Policy on Biofuels 2018. 2018 http://petroleum.nic.in/sites/default/files/biofuelpolicy2018_1.pdf.
26. Press Information Bureau, Ministry of Agriculture & Farmers Welfare, Government of India. Union Ministry for Agriculture & Farmers Welfare adopts several steps to tackle stubble burning. 2019 pib.gov.in/Pressreleaseshare.aspx?PRID=1590402 (accessed Dec 11, 2020).
27. Press Information Bureau, Ministry of Science and Technology, Government of India. India launches "Green Crackers" in its bid to curb air pollution. 2019 https://pib.gov.in/PressReleseDetailm.aspx?PRID=1587327 (accessed Dec 11, 2020).
28. Press Information Bureau, Ministry of Road Transport & Highway, Government of India. Government decides to directly shift from BS-IV to BS-VI Emission norms. 2016 https://archive.pib.gov.in/archive2/PrintRelease.aspx (accessed Dec 11, 2020).
29. Press Information Bureau, Ministry of Environment, Forest and Climate Change, Government of India. India to shift to BS VI vehicular emission norms by April next year: Union Environment Minister (7/11/2019). 2019. pib.gov.in/Pressreleaseshare.aspx?PRID=1587407 (accessed Dec 11, 2020).
30. Press Information Bureau, Ministry of Heavy Industries & Public Enterprises, Government of India. Implementation of National Electric Mobility Mission Plan. 2019 https://pib.gov.in/PressReleasePage.aspx?PRID=1577726 (accessed Dec 11, 2020).
31. Press Information Bureau, Ministry of Power, Government of India. Shri RK Singh launches National E-Mobility Programme in India; congratulates EESL for installation of 50 lakh LED street lights. 2018 https://pib.gov.in/pressreleaseshare.aspx?prid=1523123 (accessed Dec 11, 2020).
